# Loss of Flagella-Related Genes Enables a Nonflagellated, Fungal-Predating Bacterium To Strengthen the Synthesis of an Antifungal Weapon

**DOI:** 10.1128/spectrum.04149-22

**Published:** 2023-01-11

**Authors:** Dan Xiong, Zixiang Yang, Xueting He, Weimei He, Danyu Shen, Lu Wang, Long Lin, Aprodisia Murero, Tohru Minamino, Xiaolong Shao, Guoliang Qian

**Affiliations:** a College of Plant Protection, State Key Laboratory of Biological Interactions and Crop Health, Key Laboratory of Integrated Management of Crop Diseases and Pests, Nanjing Agricultural University, Nanjing, P. R. China; b Medical College, China Three Gorges University, Yichang, China; c Graduate School of Frontier Biosciences, Osaka University, Suita, Osaka, Japan; Institute of Microbiology, Chinese Academy of Sciences

**Keywords:** *L. enzymogenes*, flagella, FleQ, HSAF, evolution, environmental microbiology, gene regulation, Gram-negative bacteria

## Abstract

Loss of flagellar genes causes a nonmotile phenotype. The genus *Lysobacter* consists of numerous environmentally ubiquitous, nonflagellated bacteria, including Lysobacter enzymogenes, an antifungal bacterium that is beneficial to plants. *L. enzymogenes* still has many flagellar genes on its genome, although this bacterium does not engage in flagella-driven motility. Here, we report that loss of certain flagellar genes allows *L. enzymogenes* to strengthen its evolutionarily gained capacity in fungal killing. To clarify why this bacterium loses flagellar genes during the evolutionary process, we cloned several representative flagellar genes from Xanthomonas oryzae, a flagellated, phylogenetically related species of *Lysobacter*, and introduced them individually into *L. enzymogenes* to mimic genomic reacquisition of lost flagellar genes. Heterogeneous expression of the three *X. oryzae* flagellar structural genes (*Xo*-*motA*, *Xo*-*motB*, *Xo*-*fliE*) and one flagellar regulatory gene (*Xo*-*fleQ*) remarkably weakened the bacterial capacity to kill fungal pathogens by impairing the synthesis of an antifungal weapon, known as the heat-stable antifungal factor (HSAF). We further investigated the underlying mechanism by selecting Xo-FleQ as the representative because it is a master transcription factor responsible for flagellar gene expression. Xo-FleQ inhibited the transcription of operon genes responsible for HSAF synthesis via direct binding of Xo-FleQ to the promoter region, thereby decreasing HSAF biosynthesis by *L. enzymogenes*. These observations suggest a possible genome and function coevolution event, in which an antifungal bacterium deletes certain flagellar genes in order to enhance its ability to kill fungi.

**IMPORTANCE** It is generally recognized that flagellar genes are commonly responsible for the flagella-driven bacterial motility. Thus, finding nonflagellated bacteria partially or fully lost flagellar genes is not a surprise. However, the present study provides new insights into this common idea. We found that loss of either certain flagellar structural or regulatory genes (such as *motA*, *motB*, *fliE*, and *fleQ*) allows a nonflagellated, antifungal bacterium (*L. enzymogenes*) to stimulate its fungal-killing capacity, outlining a genome-function coevolution event, where an antifungal bacterium “smartly” designed its genome to “delete” crucial flagellar genes to coordinate flagellar loss and fungal predation. This unusual finding might trigger bacteriologists to reconsider previously ignored functions of the lost flagellar genes in any nonflagellated, pathogenic, or beneficial bacteria.

## INTRODUCTION

The bacterial flagellum is a surface-attached macromolecular nanomachine responsible for cell locomotion, allowing bacteria to seek benefits and avoid harm (1). The flagellum also plays important roles in virulence and competition for bacteria, which has been well studied in pathogenic flagellated bacteria, such as Pseudomonas aeruginosa, *Salmonella* spp., and Escherichia coli (2). The flagellar structure is composed of at least three parts as follows: the basal body, which acts as an ion-driven rotary motor; the hook, which functions as a universal joint to transmit torque produced by the motor to the filament; and the filament, which works as a helical propeller to produce thrust to push the cell body forward (3).

The basal body is composed of the C ring (FliG, FliM, FliN), MS ring (FliF), P ring (FlgH), L ring (FlgI), and the rod in most Gram-negative bacteria. The basal body also contains the flagellar type III secretion system (FT3SS) that is required for construction of the flagellum on the cell surface (4). The MS ring is composed of 34 copies of the FliF subunit and is not only for the mounting platform for the C ring but is also a housing for the FT3SS (5). The C ring is formed on the cytoplasmic face of the MS ring and acts not only as part of a rotor but also a switching device that changes the rotational direction of the flagellar motor (6–8). The LP ring complex shows 26-fold rotational symmetry and acts as a molecular bushing of the flagellar motor (9). The rod is a drive shaft consisting of the FlgB, FlgC, FlgF, FlgG, and FliE subunits (10–13). The stator complex (MotAB or PomAB) self-assembles around the MS-C ring complex and acts as a transmembrane ion channel that conducts ions such as protons (H^+^) and sodium ions (Na^+^) to generate rotational force by its interactions with FliG in the C ring (14). The hook protein (FlgE) directly assembles at the tip of the rod with the help of the hook cap (FlgD) and assembles into the hook structure (15). Upon completion of hook assembly, the hook cap is replaced by FlgK (16), and FlgK and FlgL self-assemble into the junction structure connecting the hook and filament (17, 18). Flagellin molecules assemble into the long helical filament with the help of the filament cap (FliD), which is located at the growing end of the flagellar filament (19).

Flagellar assembly is a complicated process involving more than 50 genes responsible for flagellar formation and function in Pseudomonas aeruginosa (20, 21), *Salmonella* spp., and Escherichia coli (22, 23). The hierarchy of flagellar gene expression exactly parallels the flagellar assembly process, and FT3SS coordinates flagellar gene expression with assembly. Flagellar gene expression is well controlled by a variety of transcriptional factors, including the master flagellar gene regulator, FleQ, that acts as a transcription factor responsible for flagellar gene expression and flagellar assembly in P. aeruginosa (24, 25). FleQ is a sigma factor 54/70-dependent transcription activator, belonging to the NtrC subfamily (24). It contains an N-terminal FleQ domain, known as a signal input domain that can receive signal molecules (such as c-di-GMP); a central AAA^+^ ATPase domain that is essential for ATP binding and hydrolysis and is also essential for the interaction with RpoN; and a C-terminal helix-turn-helix DNA-binding domain that is responsible for the binding to a promoter of target genes, thereby regulating their transcriptions (24). FleQ is at the top of the hierarchy of the flagellar regulon (20). FleQ can activate the transcription of class II genes encoding components of the flagellar basal body, motor, and export apparatus, thereby promoting flagellar assembly and flagella-dependent swimming motility (26). In Pseudomonas aeruginosa, flagellar gene transcription activation could be inhibited upon FleQ binding to c-di-GMP, a ubiquitous bacterial nucleotide second messenger (27). Moreover, FleQ also appears to bind to a series of promoters of exopolysaccharide (EPS) production genes to suppress EPS production, and the binding of c-di-GMP to FleQ relives such repressions, thereby promoting EPS production and biofilm formation (27). RpoN is an alternative sigma factor and induces the transcription of class II and class III genes in the Pseudomonas aeruginosa flagellar regulon (20). However, RpoN requires FleQ to recognize the class II promoters, and so FleQ is known as a master transcription regulator located at the top of the four flagellar gene regulatory cascades (20, 24). FleQ homologs are present in all *Pseudomonas* species, *Vibrio* species, and numerous flagellated *Gammaproteobacteria*, including the *Xanthomonas* genus (28, 29). In the prototypical *Enterobacteriaceae* models (E. coli and *Salmonella* spp.), the *flhD* and *flhC* genes form an operon that is at the top of the transcriptional hierarchy of the flagellar regulon, and FlhC and FlhD act as a transcriptional activator to induce transcription from class II promoters (30). The FlhD and FlhC homologs are absent in P. aeruginosa and Xanthomonas oryzae. Instead of FlhD/FlhC, FleQ acts as a master regulator to regulate the flagellar regulatory system in P. aeruginosa and *X. oryzae* (20, 28, 31).

As a general concept, loss of flagellar structural and/or regulatory genes results in a nonmotile phenotype during genome evolution. This concept is adopted by numerous nonflagellated bacteria, such as *Lysobacter*, Buchnera aphidicola, Myxococcus xanthus, and Stigmatella aurantiaca (32–35). Members of the genus *Lysobacter* are environmentally ubiquitous, and most of them are nonflagellated. These bacteria are common inhabitants of agricultural soils, and they are characterized by the ability to prey on other microorganisms through production of abundant antibiotics and lytic enzymes (36–38). Among them, Lysobacter enzymogenes OH11 (hereafter referred as to OH11) is one of the representative species of this genus, and it is a plant-beneficial bacterium protecting plants from fungal pathogen infection by producing an antifungal antibiotic, known as the heat-stable antifungal factor (HSAF) (39). HSAF is a macrocyclic lactam compound with a unique tricyclic skeleton. It is attached to a 16-circolactam, and another serine residue is embedded in the macro-ring (40). In targeting filamentous fungi, HSAF has a unique mechanism of action; it inhibits the filamentous fungal mycelia growth by disrupting the biosynthesis of fungal membrane-associated sphingolipids (41). OH11 is also a representative species of nonflagellated *Lysobacter* members, and so this species shows no flagella-dependent motility (42, 43). Alternatively, we have previously shown that OH11 exhibits a type IV pilus (T4P)-driven twitching motility over solid agar surfaces, which is proposed to help this bacterium to move toward nearby fungal pathogens and kill them by secreting HSAF and abundant lytic enzymes (44–49).

OH11 still has genes encoding components of the FT3SS on its genome (48). The FT3SS is composed of a H^+^-driven export gate complex formed by five transmembrane proteins, FlhA, FlhB, FliP, FliQ, and FliR, and a cytoplasmic ATPase ring complex consisting of FliH, FliI, and FliJ (50, 51). It has been reported that these FT3SS genes are required not only for T4P-mediated twitching motility in OH11 but also for the secretion of antifungal toxins by OH11 (48). These findings not only highlight the biological significance of the remaining flagellar structural genes in the nonflagellated *Lysobacter* but also motivate us to question whether flagellar genes lost in the genome are only responsible for the loss of flagella-driven locomotion. Here, we have hypothesized that the loss of flagellar genes may facilitate nonflagellated *Lysobacter* members to effectively produce its evolutionarily gained traits, such as the twitching behavior and the ability to predate other microorganisms. To clarify this hypothesis, we selected OH11 as a working model. We first randomly cloned seven crucial flagellar-related genes (*fleQ*, *motA*, *motB*, *fliE*, *fliG*, *flgL*, and *fliD*) from Xanthomonas oryzae PXO99A, a flagellated, phylogenetically close species of *Lysobacter*. In combination of genetics, biochemistry, and biochemical approaches, we showed that heterogeneous expression of three *X. oryzae* flagellar structural genes (*Xo-motA*, *Xo-motB*, and *Xo-fliE*) or the flagellar master regulatory gene (*fleQ*) in OH11 did not affect its twitching behavior but remarkably weakened this antifungal bacterium to kill fungal pathogens by impairing the synthesis of the antifungal factor, HSAF. We further observed that the ectopic expression of *Xo-fleQ* in OH11 inhibited HSAF production by directly binding to the promoter of the HSAF biosynthesis operon, resulting in suppression of the transcription of this operon. Our findings uncover that the loss of flagella-related genes not only accounts for the loss of flagella-dependent motility of OH11 but also enables this nonflagellated bacterium to strengthen its fungal-killing capacity, expanding our current understanding about the genomic loss of flagellar genes from its general concept to a distinct discovery.

## RESULTS

### Heterogeneous expression of three *Lysobacter* lost flagellar structural genes in *L. enzymogenes* impairs fungal-killing ability by blocking the antifungal HSAF synthesis.

**FIG 1:**
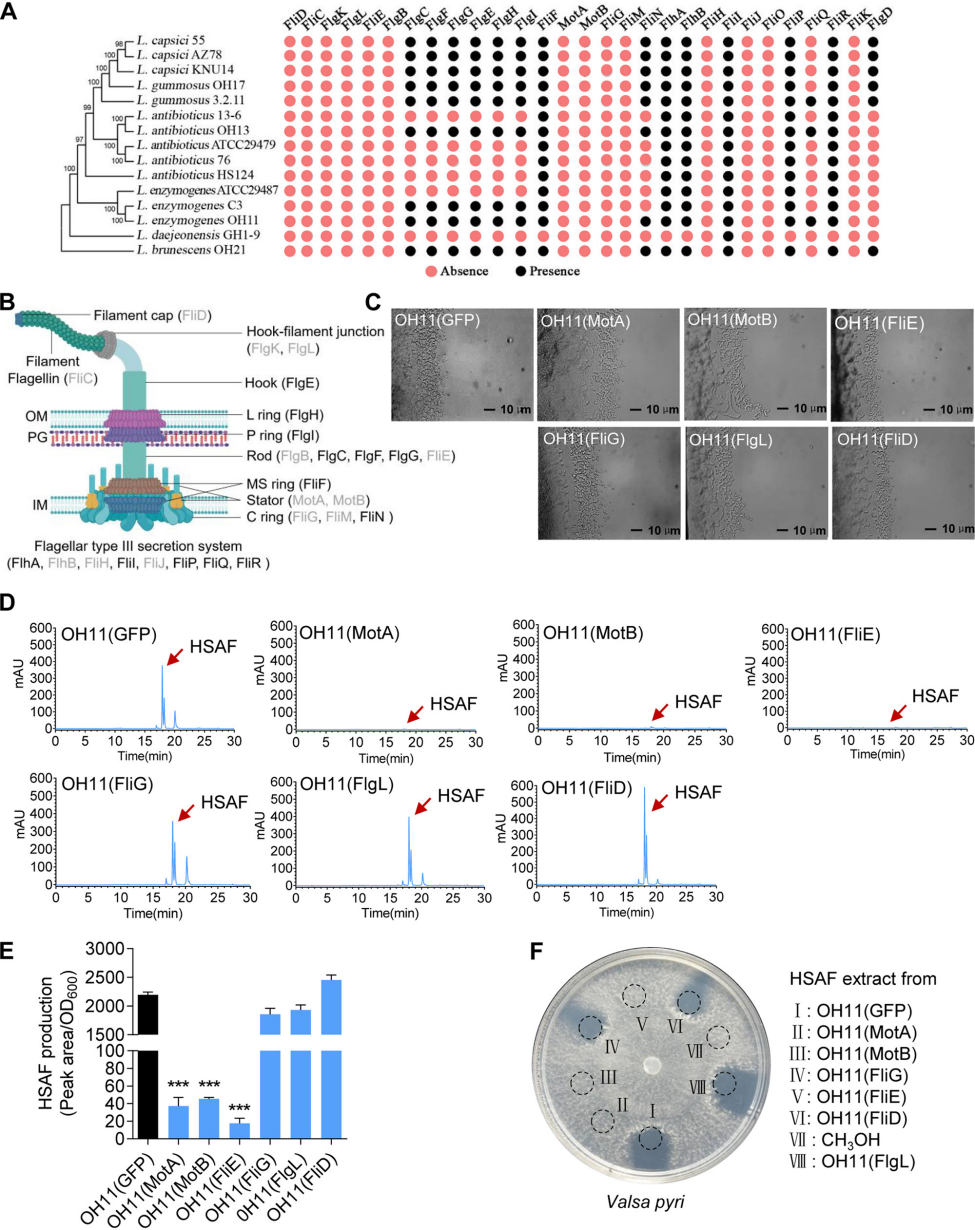
Heterogeneous expression of flagella structure genes *motA*, *motB*, and *fliE* reduced the antibiotic HSAF production in *L. enzymogenes* OH11. (A) BLAST analyzed the presence and absence of flagellar assembly genes in several *Lysobacter* genomes as represented by filled black circles and filled red circles, respectively. The flagellar genes of Xanthomonas oryzae pv. *oryzae* PXO99 were used as reference. (B) Schematic diagram of the flagellar structure of Xanthomonas oryzae pv. *oryzae* PXO99. Genes with gray indicate their absence in the genome of OH11. (C) Observation of twitching motility by heterogeneous expression of the *X. oryzae* flagellar structural genes in OH11. In 1/10 tryptic soy broth (TSB) agar medium, control OH11(GFP) exhibits twitching motility, as evidenced by the appearance of mobile cells at the colony margin, while the overexpression strains, including the Xo-MotA, Xo-MotB, Xo-FliE, Xo-FliG, Xo-FliD, and Xo-FlgL strains, did not affect the twitching motility of OH11 compared with that of the control. (D) Representative results of HPLC chromatogram showing HSAF production from OH11(GFP) and OH11(Xo-MotA), OH11(Xo-MotB), OH11(Xo-FliE), OH11(Xo-FliG), OH11(Xo-FlgL), and OH11(Xo-FliD). Red arrow indicates peak of HSAF. The assays were done three times with similar results. (E) Quantification of HSAF levels produced by the OH11(GFP) and OH11(Xo-MotA), OH11(Xo-MotB), OH11(Xo-FliE), OH11(Xo-FliG), OH11(Xo-FlgL), and OH11(Xo-FliD) as measured by HPLC. HSAF amounts (*y* axis) expressed as peak intensities from the HPLC chromatogram per unit of bacterial optical density, OD_600_ (62). The results are expressed as mean ± standard deviation (SD). ***, *P *< 0.001. (F) Antifungal activity assay based on HSAF yield against *V. pyri* in the 1/10 TSB agar plate. Ethyl acetate extractive fermentation for 24 h 1/10 TSB and CH_3_OH dissolved. OH11(GFP), control; OH11(Xo-FleQ), expression of Xo-FleQ in OH11; OH11(Xo-FliD), expression of FliD in OH11; OH11(Xo-FliE), expression of Xo-FliE in OH11; OH11(Xo-FlgL), expression of Xo-FlgL in OH11; OH11(Xo-FliG), expression of Xo-FliG in OH11; OH11(Xo-MotA), expression of Xo-MotA in OH11; OH11(Xo-MotB), expression of Xo-MotB in OH11; CH_3_OH, negative control. All experiments were repeated at least three times with similar results.

To test whether the lost flagellar genes interfere with the capabilities of OH11 to exhibit T4P-driven twitching motility over solid surfaces and/or to prey on other microorganisms, we first conducted a detailed search in the genomes of 15 *Lysobacter* species that do not produce flagella to identify what flagellar genes are missing from the *Lysobacter* genomes. As shown in Fig. 1A, we found that 14 flagellar structural genes were absent to various degrees in the *Lysobacter* genomes. The lost flagellar structural genes shared by all *Lysobacter* species encode component proteins of the flagellar core structure, including the filament cap (*fliD*), the filament (flagellin gene, *fliC*), the hook-filament junction structure (*flgK* and *flgL*), the rod (*flgB* and *fliE*), the C ring (*fliG* and *fliM*), and the stator complex (*motA* and *motB*) (Fig. 1B).

To investigate whether these *Lysobacter* lost core flagellar structural genes could connect the flagellar loss and predation-associated behaviors together, we carried out a series of genetic assays using OH11 as a working model. As an initial attempt, we randomly cloned six *Lysobacter* lost flagellar structural genes (*motA*, *motB*, *fliE*, *fliG*, *flgL*, and *fliD*) from *X. oryzae* PXO99A, a flagellated, phylogenetically close species of *Lysobacter*. Each of these six genes was fused with a C-terminal green fluorescent protein (GFP) tag and was heterogeneously expressed in OH11 transformed with plasmids in an attempt to mimic the genomic regain of these lost flagellar genes in OH11. The correct expression of each gene in OH11 was confirmed by observing the GFP signal (see Fig. S1A in the supplemental material). This step finally resulted in the generation of six OH11 derivative strains, including OH11(*Xo-motA*), OH11(*Xo-motB*), OH11(*Xo-fliE*), OH11(*Xo-fliG*), OH11(*Xo-flgL*), and OH11(*Xo-fliD*). Monitoring bacterial growth in liquid LB broth revealed that OH11 expressing each gene from the plasmid did not affect the growth rate compared to that of the GFP control (Fig. S1B).

Next, we tested whether the expression of each of the heterogeneous flagellar structural genes in OH11 affects the T4P-driven twitching motility that acts as one of the fungal predation-associated behaviors. As shown in Fig. 1C, we found that OH11 expressing *Xo-motA*, *Xo-motB*, *Xo-fliE*, *Xo-fliG*, *Xo-flgL*, or *Xo-fliD* displayed a twitching phenotype in a similar way to the wild-type strain possessing a GFP control. We then tested whether the antifungal HSAF would be changed upon OH11 expressing each of the six flagellar genes because HSAF is the major antifungal weapon required for this bacterium to kill fungi during its fungal predation process. We quantified the amount of HSAF production of the above six ectopically expressed strains using high-performance liquid chromatography (HPLC). When either Xo-MotA, Xo-MotB, or Xo-FliE were expressed in OH11, they suppressed HSAF production compared to that of the GFP control. In contrast, neither Xo-FliG, Xo-FlgL, nor Xo-FliD affected HSAF production by OH11 (Fig. 1D and E). To validate the above findings, we performed antifungal assays on agar plates using the filamentous fungal pathogen, Valsa pyri as an indicator (52–54). According to our earlier report (55), *Valsa pyri* is sensitive to HSAF; thus, we selected this laboratory available filamentous fungal pathogen causing pear valsa canker as an indicative fungus to carry out antifungal assays. In agreement with HSAF measurements, the HSAF extracts from OH11 expressing *Xo-motA*, *Xo-motB*, or *Xo-fliE* failed to inhibit *V. pyri* growth in the plating assay, in contrast to the HSAF extract from OH11 expressing GFP, *Xo-fliG*, *Xo-flgL*, or *Xo-fliD* (Fig. 1F). These results indicate that loss of *motA*, *motB*, or *fliE* enhances the capability of OH11 to kill fungi, providing a previously unknown linker connecting the genetic trait of the flagellar loss and the ecological trait of fungal predation.

### Ectopic expression of the master flagellar regulator FleQ gene in *L. enzymogenes* also weakens HSAF-dependent fungi-killing activity.

**FIG 2:**
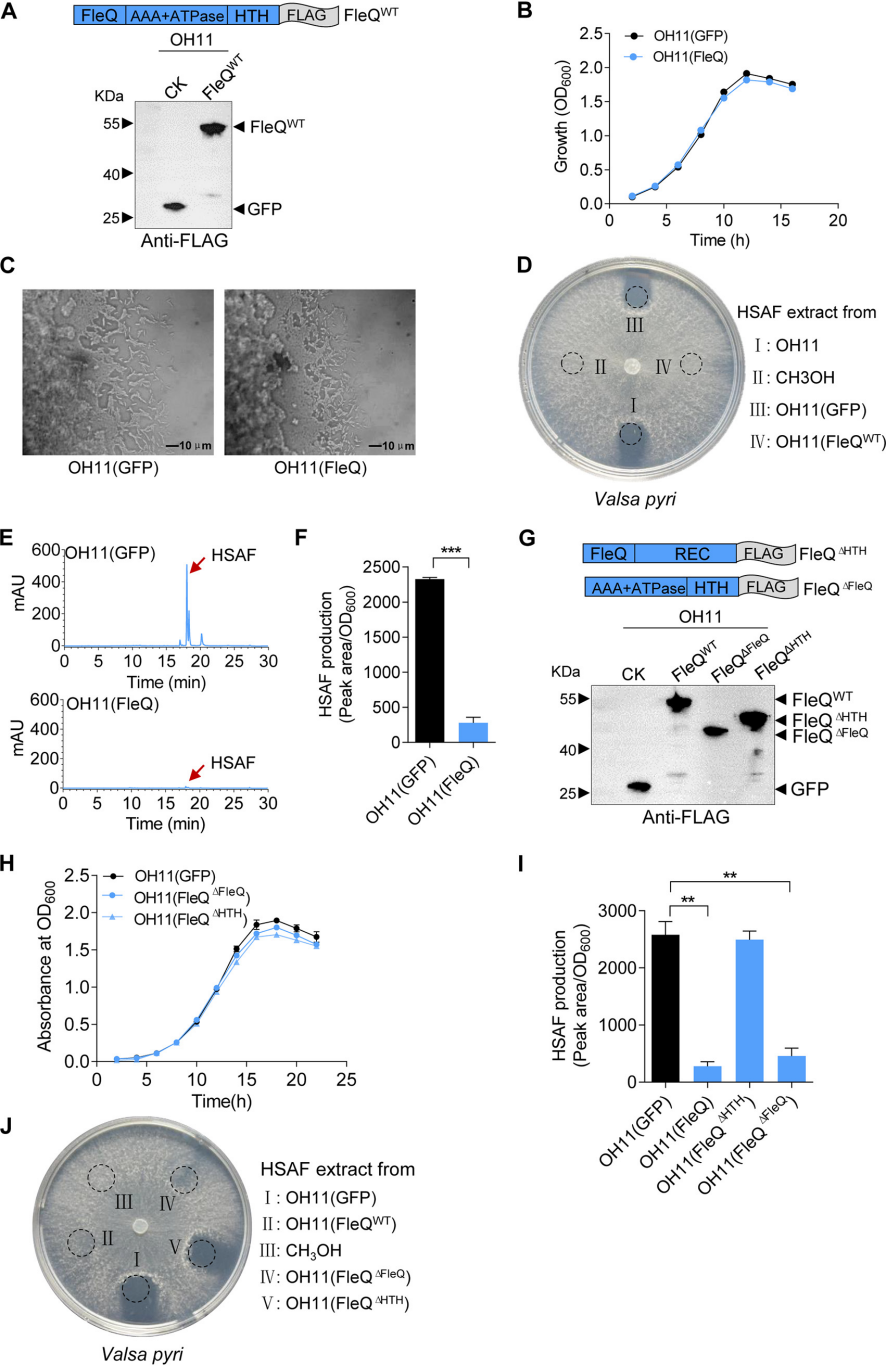
Ectopic expression of Xo-FleQ in OH11 inhibited the HSAF-based antifungal activity of *V. pyri*. (A) Western blot showing Xo-FleQ protein expression. The coding gene of Xo-FleQ was fused with a Flag-tag and introduced into wild-type OH11. (B) The expression of Xo-FleQ does not perturb the growth of *L. enzymogenes* in the 1/10 TSB broth. (C) In 1/10 tryptic soy broth (TSB) agar medium, CK OH11(GFP) exhibit twitching motility, as evidenced by the appearance of mobile cells at the colony margin, FleQ did not affect the twitching motility of OH11 compared with the control. (D) Antifungal activity assay based on HSAF yield against *V. pyri* in the 1/10 TSB agar plate. Ethyl acetate extractive fermentation for 24 h 1/10 TSB and CH_3_OH dissolved. OH11, wild-type strain; OH11(GFP), control; OH11(Xo-FleQ), expression of Xo-FleQ in OH11; CH_3_OH, negative control. (E) Representative results of HPLC chromatogram showing HSAF production from OH11(GFP) and OH11(Xo-FleQ). Red arrow indicates peak of HSAF. The assays were done three times with similar results. Strains were cultivated in 1/10 TSB, and cells were collected at an OD_600_ of 1.0. The results are expressed as mean ± SD. ***, *P *< 0.001. (F) Quantification of HSAF levels produced by the OH11(GFP) and OH11(Xo-FleQ) as measured by HPLC. HSAF amounts (*y* axis) expressed as peak intensities from the HPLC chromatogram per unit of bacterial optical density, OD_600_ (62). (G) Western blot showing Xo-FleQ, Xo-FleQ^ΔHTH^, and Xo-FleQ^ΔFleQ^ protein expression. The coding gene of Xo-FleQ was fused with a Flag-tag and introduced into wild-type OH11. Strains were cultivated in 1/10 TSB, and cells were collected at an OD_600_ of 1.0. (H) The expression of Xo-FleQ^ΔHTH^ and Xo-FleQ^ΔFleQ^ does not perturb the growth of *L. enzymogenes* in the 1/10 TSB broth. The results are expressed as mean ± SD. (I) Quantification of HSAF levels produced by the OH11(GFP), OH11(FleQ), OH11(FleQ^ΔHTH^), and OH11(FleQ^ΔFleQ^) as measured by HPLC. HSAF amounts (*y* axis) expressed as peak intensities from the HPLC chromatogram per unit of bacterial optical density (OD_600_) (62). (J) Antifungal activity assay based on HSAF yield against *V. pyri* in the 1/10 TSB agar plate. Ethyl acetate extractive fermentation for 24 h 1/10 TSB and CH_3_OH dissolved. OH11(GFP), control; OH11(FleQ), expression of Xo-FleQ in OH11; OH11(FleQ^ΔHTH^), expression of Xo-FleQ^ΔHTH^ in OH11; OH11(FleQ^ΔFleQ^), expression of Xo-FleQ^ΔFleQ^ in OH11; CH_3_OH, negative control. All experiments were repeated at least three times with similar results.

The above findings motivated us to explore whether the *Lysobacter* lost flagellar regulatory genes could also contribute to the bacterial capacity in fungal killing. To facilitate this investigation, we selected the best-studied master flagellar gene regulator FleQ because it is at the top of the hierarchy in the flagellar regulon in P. aeruginosa and so induces all genes responsible for flagellar assembly, motility, and chemotaxis (24, 31). We cloned the *X. oryzae fleQ* (*Xo-fleQ*) fused with a C-terminal FLAG tag by PCR and introduced the plasmid-borne *Xo-fleQ-FLAG* into OH11, followed by Western blotting using the anti-FLAG antibody to validate its correct expression (Fig. 2A). OH11 expressing the heterogeneous Xo-FleQ displayed no growth or twitching defects compared to those of OH11 expressing GFP (Fig. 2B and C). However, the heterogeneous expression of Xo-FleQ significantly impaired HSAF production by OH11, thereby reducing HSAF-mediated antifungal activity (Fig. 2D to F).

FleQ is predicted to possess three domains, an N-terminal FleQ domain, a middle AAA^+^ ATPase domain, and a C-terminal HTH (helix-turn-helix) DNA binding domain (Fig. 2A). To identify which domain is responsible for a significant decrease in HSAF production by OH11, we generated two FleQ truncation variants, namely, Xo-FleQ^ΔFleQ^ lacking the N-terminal FleQ domain and Xo-FleQ^ΔHTH^ lacking the C-terminal HTH domain, respectively. We individually introduced a plasmid encoding either Xo-FleQ^ΔFleQ^-FLAG or Xo-FleQ^ΔHTH^-FLAG genes into OH11, and then their ectopic expression was confirmed by Western blotting using anti-FLAG antibody (Fig. 2G). Neither Xo-FleQ^ΔFleQ^-FLAG nor Xo-FleQ^ΔHTH^-FLAG affected the cell growth at all (Fig. 2H). The HPLC-based HSAF quantification assays showed that removal of the C-terminal HTH domain from Xo-FleQ restored HSAF production to the wild-type level, whereas that of the N-terminal FleQ domain did not (Fig. 2I), indicating that the C-terminal HTH domain of FleQ directly inhibits HSAF production by OH11. In agreement with HSAF measurements, the HSAF extract from OH11 expressing the FleQ^ΔFleQ^ gene still efficiently inhibited *V. pyri* growth in the plating assay, while OH11 possessing the FleQ^ΔHTH^ gene did not (Fig. 2J). These results collectively suggest that the loss of the flagellar regulator FleQ gene is also beneficial for nonflagellated *L. enzymogenes* to strengthen the HSAF-based fungal killing capacity.

### Xo-FleQ directly bind to the promoter of the operon responsible for HSAF biosynthesis in *L. enzymogenes*, thereby repressing its transcription.

**FIG 3:**
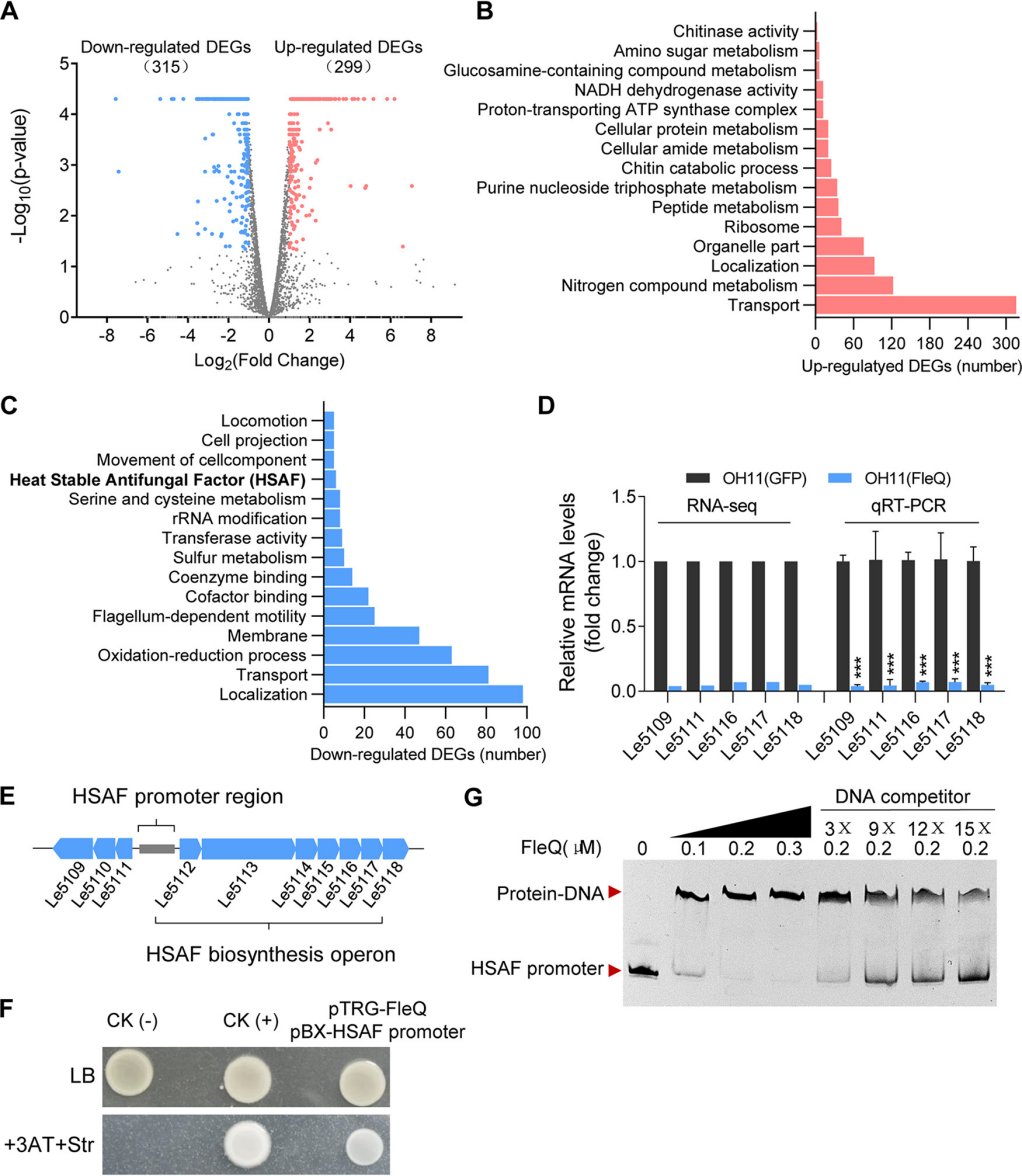
RNA-seq revealed that Xo-FleQ directly regulated the transcription of HSAF biosynthesis operon genes. (A) Volcano plot indicating differentially expressed genes (DEGs) between WT_OH11_/GFP and WT_OH11_/FleQ cells as analyzed by RNA-seq. (B) The top 15 most significantly enriched pathways from the KEGG pathway analysis with upregulated DEGs. The pathways shown were determined under a threshold FDR of <5%. (C) The top 15 most significantly enriched pathways from the KEGG pathway analysis with downregulated DEGs. The pathways shown were determined under a threshold FDR of <5%. (D) Transcription of open reading frames Le5109, Le5111, Le5116, Le5117, and Le5118 of the HSAF synthetic gene cluster in RNA-seq data; qRT-PCR analyses of HSAF synthesis gene cluster open reading frames, including Le5109, Le5111, Le51116, Le5117, and Le5118 mRNA quantity in the control and expression FleQ. OH11(GFP), control strain; OH11(FleQ), expression of Xo-FleQ in OH11. In all assays, average data from three experiments are presented ± SD. **, *P* < 0.01. The results are expressed as mean ± SD. ***, *P *< 0.001. (E) The HSAF biosynthetic gene cluster contains 10 open reading frames, Le5109, Le5110, Le5111, Le5112, Le5113, Le5114, Le5111, Le5116, Le5117, and Le5118. (F) The direct physical interaction between Xo-FleQ and the *lafB* promoter region was detected in E. coli using bacterial one-hybrid assay. Positive control (CK^+^), cotransformant containing pBX-R2031 and pTRG-R3133; negative control (CK^−^): cotranformant containing pBXcmT and empty pTRG. (G) The N terminus of HSAF biosynthesis operon promoter DNA fragment was labeled with FAM. The electrophoretic mobility shift assay (EMSA) experiments showing Xo-FleQ binding to HSAF promoter. The combination was further verified by the cold probe competitive reaction. All experiments were repeated at least three times, with similar results.

We next explored how Xo-FleQ inhibits HSAF synthesis by OH11. Because FleQ is a transcription factor, we performed a genome-wide transcriptome sequencing assay (RNA-seq). The OH11(GFP) and OH11(FleQ) cells grew in the HSAF-producing medium (1/10 tryptic soy broth [TSB]) until the optical density at 600 nm (OD_600_) had reached approximately 1.0, followed by RNA-seq. The RNA-seq data showed that OH11(FleQ) had a total of 299 upregulated differentially expressed genes (DEGs) and 315 downregulated DEGs compared with those of the control strain (Fig. 3A; see also Table S1 in the supplemental material). We found that the upregulated DEGs in OH11(FleQ) were involved in multiple functions, such as transport process, nitrogen compound metabolism, and localization (Fig. 3B; see also Table S1). The downregulated DEGs in OH11(FleQ) were also involved in localization, transport process, oxidation-reduction process, membrane, and HSAF biosynthesis (Fig. 3C; see also Table S1). What is of great concern to us is that nine HSAF biosynthesis related genes, including the HSAF biosynthesis operon (Le1512–1518) were significantly downregulated in OH11(FleQ) (Fig. 3D). We confirmed this finding by real-time quantitative PCR (RT-qPCR) assays (Fig. 3D). These findings collectively suggest that the transcriptional inhibition of the HSAF biosynthesis operon most likely accounts for the suppression of HSAF production by Xo-FleQ in OH11.

Since Xo-FleQ acts as a transcription factor in *X. oryzae*, we tested whether it could bind to the promoter region of the HSAF biosynthesis operon, resulting in a direct transcription regulation of this operon. We first tested this hypothesis via the bacterial one-hybrid assay. The results did produce a positive signal because we indeed found that Xo-FleQ directly bound to the promoter of the HSAF biosynthesis operon (Fig. 3E to G). Via electrophoretic mobility shift assay (EMSA), we also found that the FleQ-His formed a protein-DNA complex with the HSAF promoter probe labeled with biotin and that the addition of the unlabeled probe could competitively inhibit this protein-DNA complex formation in a dose-dependent manner (Fig. 3G). Taken all together, we suggest that removal of FleQ from the genome not only correlates with the trait that makes OH11 a nonflagellated bacterium but also allows this bacterium to relieve the inhibition of HSAF biosynthesis by FleQ.

## DISCUSSION

It is generally recognized that flagellar genes are commonly responsible for flagella-driven bacterial motility under various environmental conditions (56–58). Thus, finding that nonflagellated bacteria partially or fully lost flagellar genes is not a surprise. However, the present study provides new insights into this common idea. We found that loss of either certain flagellar structural or regulatory genes allows a nonflagellated, antifungal bacterium to stimulate its fungal killing capacity, outlining a genome-function coevolution event, where an antifungal bacterium “smartly” designed its genome to “delete” crucial flagellar genes to coordinate flagellar loss and fungal predation. This unusual finding might trigger bacteriologists to reconsider the previously ignored functions of the lost flagellar genes in any nonflagellated, pathogenic, or beneficial bacteria.

As a representative member of nonflagellated bacteria, the genus *Lysobacter* comprises various plant-beneficial species, with OH11 as one of the representative species acting as a natural predator of fungal pathogens (59–62). It moves toward to nearby fungi via T4P-driven twitching motility over solid surfaces to kill them through the production of abundant lytic enzyme/toxins and the antifungal antibiotic HSAF (38, 40). We have previously shown that the retained flagellar type III secretion system (FT3SS) is functional in nonflagellated Lysobacter enzymogenes OH11 so that the hook-capping protein FlgD is secreted into the culture media (48). It has been reported that FliE is the first export substrate secreted by the FT3SS (63) and that interactions of FliE with FliP and FliR, which are export gate proteins of the FT3SS, fully open the exit gate of the protein export channel in the FT3SS, allowing other flagellar building blocks to diffuse down the central channel of the growing flagellar structure (64). Assuming that certain positive regulators, which may be under the control of FleQ derived from Xanthomonas oryzae (Xo-FleQ) and facilitate the production of an antifungal weapon (HSAF) by the OH11 strain, may be secreted via the FT3SS, it is speculated that the expression of Xanthomonas oryzae FliE (Xo-FliE) would fully open the export channel of the FT3SS through interactions of Xo-FliE with FliP and FliR, allowing the positive regulators to exit the cell and suppressing HSAF production. MotA and MotB form a transmembrane proton channel complex in the cytoplasm that acts as a stator unit of the flagellar motor that couples inward-directed proton flow with torque generation. Because proton motive force (PMF) across the cytoplasmic membrane is the primary driving force for flagellar protein export by the FT3SS (65), the expression of either Xo-MotA or Xo-MotB may affect the level of PMF across the cytoplasmic membrane, thereby reducing the protein export activity of the FT3SS in the OH11 strain. As a result, certain negative regulators, which are also under the control of Xo-FleQ and may suppress HSAF production, could be accumulated in the cytoplasm, thereby inhibiting HSAF production. FlgK and FlgL together form the junction structure connecting the hook and filament in flagellated bacteria and do not contribute to flagellar protein export by the FT3SS (18, 66). Although the *Salmonella* FT3SS requires FliG to exert the full protein export activity because the C-ring composed of FliG, FliM, and FliN provides the binding site for the cytoplasmic ATPase ring complex (67), the FT3SS is still functional even in the absence of the FliG homolog in the OH11 strain. Therefore, we speculate that neither Xo-FliG, Xo-FlgK, nor Xo-FlgL affect HSAF production. However, it was honestly unclear why heterogeneous expression of the *Xo-motA*, *Xo-motB*, or *Xo-fliE* gene in *L. enzymogenes* OH11 reduces the production of the antifungal HSAF, whereas heterogeneous expression of either *fliG*, *flgL*, or *fliD* does not. However, our data clearly support our conclusion that loss of certain flagella-related genes placed under the control of FleQ strengthens HSAF production in nonflagellated *Lysobacter*. However, this only provides one available phenotype/function that is controlled by the lost flagella-related genes in *Lysobacter*. We could not eliminate the possibility that the lost flagella-related genes (i.e., *fliG*, *flgL*, and *fliD*) in nonflagellated *Lysobacter* might have other HSAF-unrelated/unknown cellular functions since multiple *Lysobacter* members presented in Fig. 1 did not seem to produce HSAF, as their genomes lack the HSAF biosynthetic gene cluster (see Table S2 in the supplemental material). Thus, in addition to the retained FT3SS, the loss of flagellar structural genes seems to act as another *Lysobacter* genomic-evolution characteristic linking the flagellar loss and fungal killing together, both of which are well-known traits for *Lysobacter* members in taxonomy and ecology.

It is also noteworthy that the loss of flagellar regulatory genes, represented by FleQ, serves as an additional genomic-designing strategy favoring the nonflagellated *L. enzymogenes* to strengthen its antifungal weapon HSAF to efficiently kill fungi. FleQ, indicated by its name, is a well-studied, master transcription factor that globally controls flagellar gene expression in P. aeruginosa (68). FleQ also acts as the receptor of c-di-GMP, a nucleotide second messenger ubiquitous in bacterial cells (69). In the human pathogenic P. aeruginosa, elevated c-di-GMP levels can reduce flagella-dependent swarming motility and promote biofilm formation, thereby switching the P. aeruginosa lifestyle from the motile planktonic state to the sessile state, which facilitates bacterial infection (68–71). In this bacterium, FleQ binding to c-di-GMP can switch to a c-di-GMP-dependent lifestyle (27, 72, 73). We found that the regain of FleQ in *L. enzymogenes* by heterogeneous expression remarkably impairs the bacterial ability to kill fungi. This effect is due to the direct regulation of FleQ on HSAF production, where the ectopically expressed FleQ could directly bind to the promoter of the HSAF biosynthesis operon, thereby suppressing the transcription of this operon. This finding provides one of the reasonable clues explaining why *L. enzymogenes* prefers to delete FleQ during genomic evolution. Another reasonable explanation is that OH11 has evolved an intrinsic regulatory network regulating HSAF biosynthesis, where the transcription factor Clp is a key member. Like FleQ, Clp also functions as the receptor protein of c-di-GMP (74, 75). Clp could directly bind to the HSAF biosynthesis operon to stimulate the HSAF biosynthesis, and the binding of c-di-GMP to Clp reduces the binding affinity Clp for DNA, thereby suppressing HSAF synthesis in OH11 (60, 62). Thus, we proposed that the loss of FleQ could relieve the interference of FleQ on HSAF synthesis and ensures the optimized operation of the native Clp-dependent network.

In addition, our data show that loss of the flagellar motor proteins without DNA binding proteins, such as MotA, MotB, and FliE, also inhibit HSAF production and antifungal activity. That a specific protein without a DNA binding domain could affect downstream gene expression in bacterial cells is not a surprise because such proteins could do this by binding downstream transcription factors or regulatory small molecules (i.e., c-di-GMP). In *L. enzymogenes* OH11, a set of proteins has been identified as HSAF biosynthesis regulators by our laboratory, including transcription factors Clp (62), LysR (76), and MarR (77); c-di-GMP binding protein CdgL (78); as well as the c-di-GMP synthesis or degradation enzymes LchD (79), LchP (62), and WspR (79). Although we do not know more details about how motor proteins affect HSAF production, the possibility that the lost flagella motor proteins (MotAB) would physically interact with *L. enzymogenes* c-di-GMP synthesis or degradation enzymes (i.e., LchD, LchP, or WspR) via transmembrane domain or utilize their cytoplasmic domains to interact with one or more transcription factors described above could not be eliminated.

**FIG 4:**
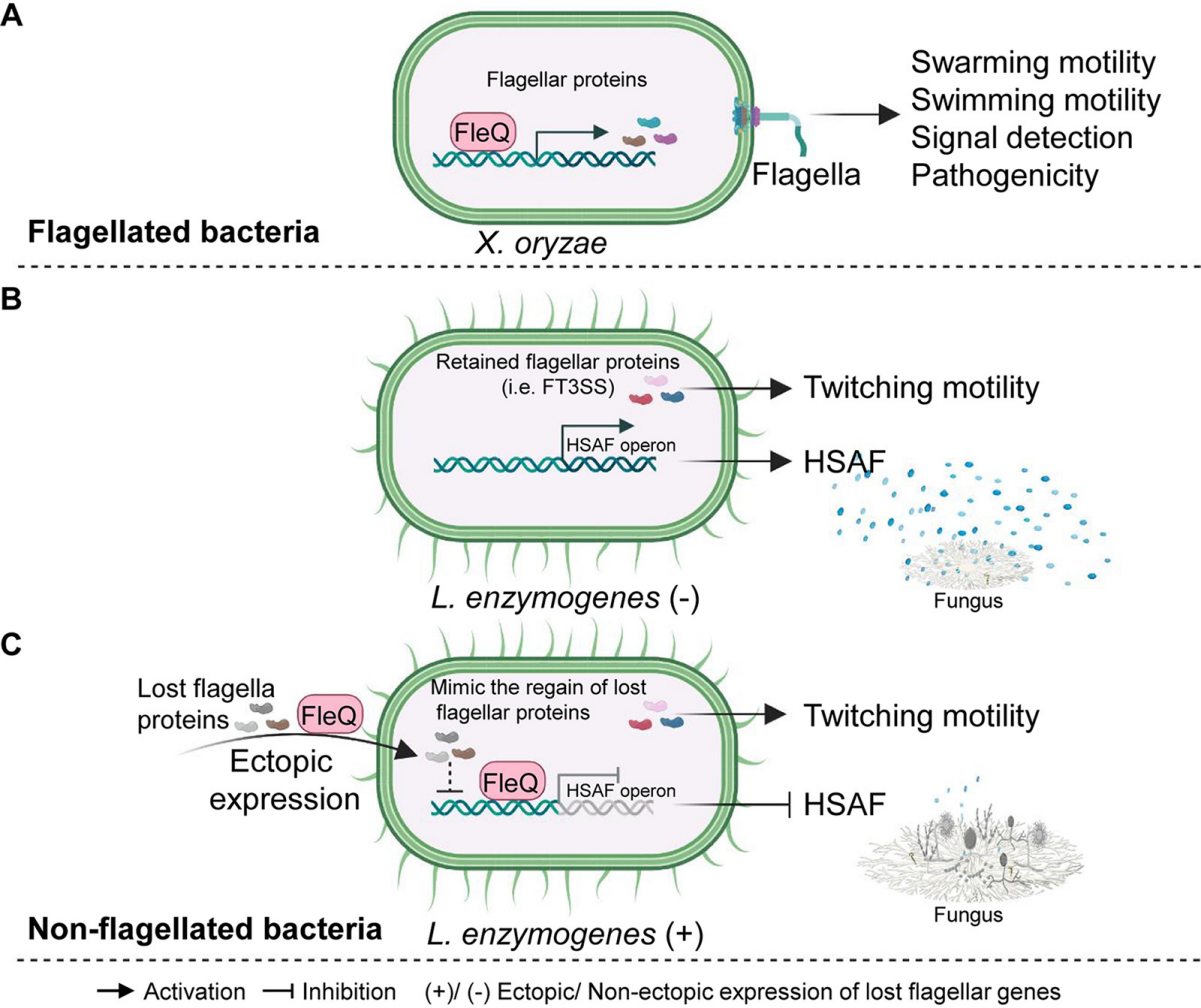
A proposed model showing the unusual effects of the retained and lost flagellar genes in the nonflagellated *L. enzymogenes*. (A) The general understanding of flagellar genes in flagellated bacteria. Flagellated bacteria, such as *Xanthomonas* spp., have complete structure of flagellar genes and regulatory systems to mediate swarming motility, signal detection, and pathogenicity. (B) The retained flagellar genes (i.e., FT3SS genes, *flhA*, *flhB* and *fliI*) in *L. enzymogenes* OH11 contribute to twitching motility on solid interfaces and could not affect production of long-arm weapon HSAF for fungal killing as reported earlier. (C) The loss of flagellar genes enables the nonflagellated *L. enzymogenes* to effectively kill fungi through the antifungal weapon, HSAF. The regain of flagellar structural or regulator genes (such as *Xo-motA*, *Xo-motB*, *Xo-fliE*, and *Xo-fleQ*) by heterogeneous expression impaired the *L. enzymogenes* capacity in fungal killing by blocking the HSAF synthesis via directly or indirectly blocking the expression of the HSAF biosynthetic gene operon.

In summary, the present study reveals that the loss of certain flagella-related genes not only provides genetic evidence explaining why *L. enzymogenes* became a nonflagellated bacterium but also favors this fungi-predating bacterium to strengthen the synthesis of an antifungal weapon (Fig. 4). Our findings might open a gate for bacteriologists to explore the biological significance of any flagellar genes that are evolutionarily lost in nonflagellated bacteria of interest.

## MATERIALS AND METHODS

### Bacterial strains, plasmids, and growth conditions.

The strains and plasmids used in this study are shown in Table S3 in the supplemental material. Escherichia coli DH5α/Top10 for plasmid construction was aerobically grown in lysogenic broth (LB) medium (10 g/L tryptone, 10 g/L NaCl, 5 g/L yeast extract, pH 7.0) at 37°C with appropriate antibiotics (50 μg/mL gentamicin [Gm]). *L. enzymogenes* OH11 and its derivative strains were cultivated in LB medium at 28°C with appropriate antibiotics (for overexpression construction, 100 μg/mL kanamycin [Km]; and for plasmid maintenance, 150 μg/mL Gm).

### HASF extraction.

HSAF extraction by high-performance liquid chromatography (HPLC) was performed as described previously (40). Briefly speaking, HSAF of *L. enzymogenes* OH11 and its derivative strains was extracted from 25 mL 1/10 TSB medium that was cultured at 28°C for 24 h with shaking at 220 rpm. We took 4 mL of fermentation broth and extracted with ethyl acetate. The HSAF was then detected and quantified via HPLC (Agilent 1260, Santa Clara, USA). Relative amounts of HSAF are expressed as peak intensity from the HPLC chromatogram per unit of OD_600_, according to earlier reports (80). Three biological replicates were used, with each run analyzed in three technical replicates.

### Antifungal activity assay.

An antifungal activity assay was conducted based on our previous studies (81). The HSAF extracted in the previous step was dissolved in methanol and filtered for sterilization. The methanol CH_3_OH was thus chosen as a negative control. Then, 3 μL HSAF extract was inoculated on the edge of 1/10 TSB agar plates that have been inoculated with *V. pyri* SXYL134 that was transferred from potato dextrose agar (PDA) medium. *V. pyri* SXYL134 is a pathogenic fungus that causes pear Valsa canker.

### Twitching motility assay.

Twitching motility assay in *L. enzymogenes* was conducted based on our previous studies (82). Briefly, the 5% TSA (tryptic soy agar) medium was poured into a 10 by 10 square petri dish, not too thick. After the medium had solidified, the medium was divided into small cubes using a sterile scalpel and placed on sterile slides. We dipped the edge of the sterilized microscope coverslips into bacterial cell suspension, then gently pressed the coverslips against the surface of the small cube medium. After keeping moist and keeping the culture at 28°C for 24 h, the bacterial edges on the slides were observed under a microscope (Olympus Corporation, Japan; Axio Observer 3, Zeiss, Germany).

### Quantitative real-time PCR.

qRT-PCR was performed as previously described (80). In simple terms, we grew the *L. enzymogenes* OH11 and its derivatives in LB medium with 50 μg/mL Km and 50 μg/mL Gm overnight. We then transferred a 1% volume of the cells to the 1/10 TSB (tryptic soy broth) medium at 28°C under shaking. When cells of strains reached an OD_600_ of 1.0 (exponential phase), we then collected the cells by centrifugation (12,000 rpm) at room temperature for total RNA extraction using the bacterial RNA kit (number AF505B; Proteinssci, Shanghai, China) following the manufacturer's instructions. RNA integrity and concentration were examined with a Thermo Scientific NanoDrop 2000 and agarose gel electrophoresis. Next, the RNA samples were supplied for cDNA synthesis using the HiScript III RT SuperMix for qPCR (+ genomic DNA [gDNA] wiper) (number R323-01; Vazyme, Nanjing, China). The primers used for qRT-PCR assays are listed in Table S4 in the supplemental material, with the 16S rRNA gene serving as an internal control as previously described (80).

### RNA-seq.

The RNA-seq assay was carried out as previously described (78). Briefly, the strains OH11(FleQ) and OH11(GFP) were grown in LB agar medium at 28°C overnight. We then transferred a 1% volume of the cells to the 1/10 TSB (tryptic soy broth) medium at 28°C under shaking. When the OD_600_ reached 1.0, the cells were collected and sent to the Beijing Genomics Institute for sequencing. Three biological replicates of each strain were used. Differentially expressed genes were determined as fold change greater than 2 with an adjusted *P* value of less than 0.05.

### Western blot analysis.

The protein samples were separated by sodium dodecyl sulfate-polyacrylamide gel electrophoresis (SDS-PAGE) and transferred to a polyvinylidene difluoride (PVDF) membrane (Millipore, USA) using a Bio-Rad semidry blotter (Bio-Rad, USA). The membranes were probed using the method previously described (55). In short, the membrane was blocked in 20 mM Tris-buffered saline with 0.05% Tween 20 (TBST) (pH 8.0) in 5% skim milk for 1 h at room temperature and then incubated with the primary antibody for 1 h or for 12 h at 4°C. Subsequently, the treated membrane was washed three times with TBST for 15 min each before incubating with a secondary antibody for 1 h at room temperature. After three more washes in TBST for 15 min each, the immune-reactive proteins could be localized. The membranes were treated with Clarity Western ECL substrate (number 170-5061; Bio-Rad, USA) for 5 min under dark conditions. The primary antibody used is monoclonal mouse anti-FLAG (number M20008S; Abmart, Shanghai, China) at a dilution of 1:5,000. The secondary antibody used is goat anti-mouse secondary antibody (number M21001L; Abmart, Shanghai, China) at a dilution of 1:5,000. Immune-reactive bands were visualized by the VIBER molecular imaging system (Fusion FX7, France).

### Protein localization and expression assay by fluorescence microscopy.

The protein localization and expression assay was conducted as previously described (83). To determine protein localization and expression of MotA, MotB, FliG, FliD, FliE, and FlgL in *L. enzymogenes* OH11. We fused green fluorescent protein (GFP) fragment sequences into the C termini of MotA, MotB, FliG, FliD, FliE, and FlgL genes, respectively, and constructed fragments into pBBRIMCS-5 plasmid using the KpnI/XbaI restriction site. The recombinant plasmid was transformed into wild-type OH11. The plasmid pBBRIMCS-5 to generate the pBBR-GFP vector was used as a negative control. The primers used for protein localization and expression assays are listed in Table S4. The resulting constructs were introduced into wild-type *L. enzymogenes* OH11. All cells with GFP fluorescence were observed by inverted fluorescence microscope (Axio Observer 3; Zeiss, Germany).

### Protein expression and purification.

Expression and purification of the His-fused FleQ was performed as described previously (49, 76). Generally speaking, FleQ was amplified by PCR with the designated primer pairs listed in Table S4. After enzyme digestion (Ndel/HindIII), this gene was cloned into the pET-30a for protein expression in E. coli strain BL21(DE3) (Table S3). The resultant strain was cultivated in LB broth (containing Km at 30 μg/mL) overnight at 37°C. Then, a total of 4 mL overnight culture was transferred into a fresh 400 mL LB medium in the presence of 30 μg/mL Km and grown at 37°C with shaking at 200 rpm until an OD_600_ of ~0.3 to ~0.6 was achieved. Subsequently, isopropyl β-d-1-thiogalactopyranoside (IPTG) (Sigma) at a final concentration of 0.5 mM was added into the culture followed by further growth at 16°C for 12 h. Then, the cells were collected by centrifugation (6,000 rpm) at 4°C and resuspended in 40 mL of phosphate-buffered saline (PBS) lysis buffer supplemented with the final concentrate of 10 mM phenylmethylsulfonyl fluoride (PMSF). The cells were lysed by a brief sonication period using a Sonifier 250 (Branson Digital Sonifier; Branson), and the crude cell extracts were centrifuged at 6,000 rpm at 4°C for 10 min. Soluble protein components were collected with His trap HP Ni* resin (GE Health), the protein solution was adsorbed by His trap HP after it passed through the column and was washed in large quantities with resuspension buffers containing 30 mM, 120 mM, and 250 mM imidazole. The eluted protein is concentrated with an ultrafiltration centrifugal tube (Millipore UFC501096) and centrifugation (6,000 rpm, 4°C, and until reduced to the desired volume). Protein purity was assessed by SDS-PAGE, and protein concentration was determined using a bicinchoninic acid (BCA) protein assay kit (Sangon Biotech).

### Electrophoretic mobility shift assay.

EMSA was performed partly referring to previous studies (78). Biotin (FAM)-labeled probes of the 343-bp HSAF promoter region were synthesized by GENEWIZ (Suzhou, China). Promotor fragment and protein extract were incubated for 30 min at ~20 to ~25°C according to the specifications of the EMSA/gel-shift binding buffer (number GS005; Beyotime). The binding mixture was loaded onto the electrophoresed polyacrylamide gel. The biotinylated DNA fragments were detected by chemiluminescence using the VIBER molecular imaging system (Fusion FX7, France).

### Bacterial one-hybrid assay.

Bacterial single hybridization was referenced to previous studies (79). *lafB* is the first gene of the HSAF biosynthetic gene operon as documented previously (84). Thus, the *lafB* promoter represents the promoter of the HSAF biosynthetic gene operon that is abbreviated as the HSAF promoter. We cloned the HSAF promoter to the pBXcmT vector as reported earlier and subjected it to the bacterial one-hybrid assay to test whether it will be directly bound by the Xo-FleQ. Xo-FleQ protein was cloned into the pTRG vector. The recombinant vector of the promoter region sequence and FleQ-PTRG were simultaneously transformed into E. coli strain XL1-Blue (Table S3). The growth of transformed E. coli was evaluated by selective medium containing 3-amino-1,2,4-triazole (3AT) and streptomycin antibiotics, and it was quickly determined whether FleQ binds directly to the promoter region (growth indicates that FleQ can binds to the promoter region).

### Data availability.

The RNA-seq raw sequence data reported in this study has been submitted to NCBI GenBank under accession number PRJNA910118.
