# Supplementary material for: Loss of Flagella-Related Genes Enables a Nonflagellated, Fungal-Predating Bacterium To Strengthen the Synthesis of an Antifungal Weapon
Source: Microbiol Spectr. 2023 Jan 11;11(1):e04149-22. doi: 10.1128/spectrum.04149-22 (PMC9927559; doi:10.1128/spectrum.04149-22)
Supplement: Supplemental file 1 — Figures S1 and Tables S1-S4. Download spectrum.04149-22-s0001.pdf, PDF file, 3.0 MB [file spectrum.04149-22-s0001.pdf]

Supplementary Information

Loss of flagella-related genes enables a non-flagellated, fungal-predating bacterium to strengthen the synthesis of an antifungal weapon

Dan Xiong[a], Zixiang Yang[a], Xueting He[a], Weimei He[a], Danyu Shen[a], Lu Wang[b], Long Lin[a], Aprodisia Murero[a], Tohru Minamino[c], Xiaolong Shao[a]*, Guoliang Qian[a]*

Containing 1 supplementary figure and 4 supplementary tables

Supplementary Figures SI Figures

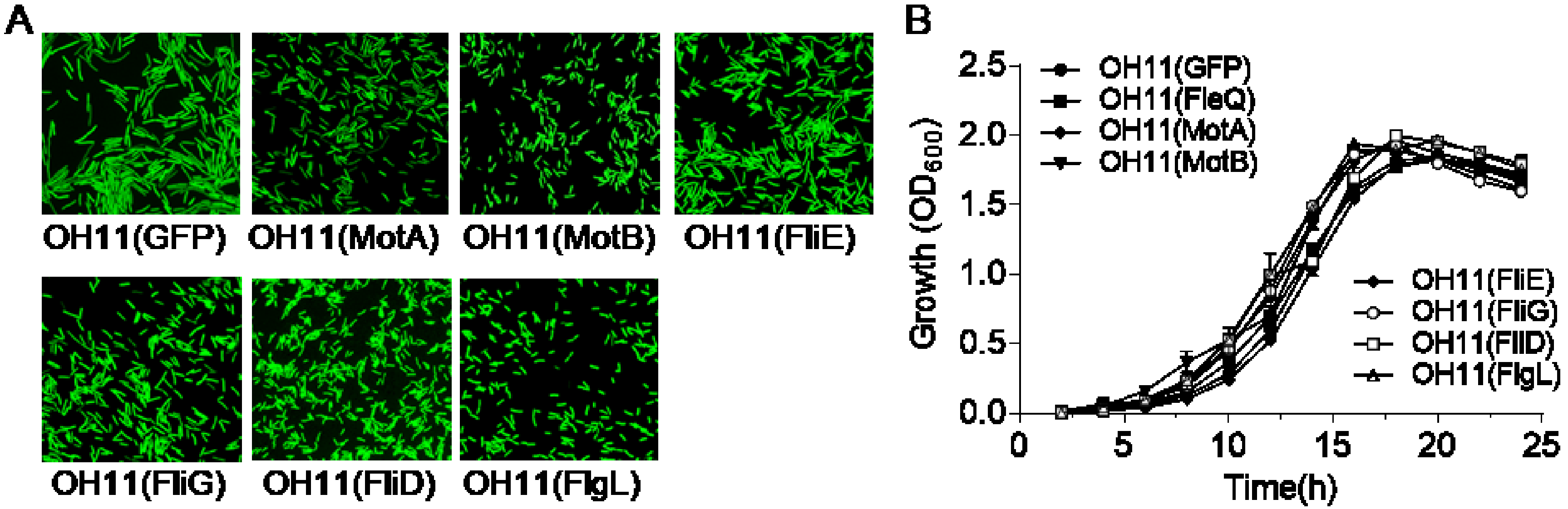

Supplementary Figure S1. Effect of MotA, MotB, FliE, FliG, FlgL, and FliD expression derived from *X. oryzae* on the growth of *L. enzymogenes* OH11. (A) Fruorescent imaging of *L. enzymogenes* OH11expressing GFP-labelled flagella structure proteins (MotA, MotB, FliE, FliG, FliD and FlgL). The coding region of MotA, MotB, FliE, FliG, FliD, and FlgL was fused with a C-terminal GFP gene. Fluorescence signals were observed by inverted fluorescence microscope (Axio Observer 3, Zeiss, Germany). **(B)** Growth curve of flagella structure genes overexpressed *L. enzymogenes* OH11 expreesing either GFP, FleQ-GFP, MotA-GFP, MotB-GFP, FliE-GFP, FliG-GFP, FliD-GFP, or FlgL-GFP. All experiment had at least three separate biological replicates.

Supplementary Tables

Table S1 RNA-seq between OH11(GFP) and OH11(FleQ)

Gene ID	GFP_ FPKM	FleQ_ FPKM	*P*_value	q_value	Fold change	Annotation
LeOH11_0915	4964.11	26.1154	0.00005	0.000658	-190.084	Beta-lactamase [Azospirillum lipoferum]
LeOH11_3603	170.93	0	0.00135	0.011151	-171.93	NA
LeOH11_3548	82.7078	1.99104	0.00005	0.000658	-41.54	inner membrane protein CreD [Collimonas fungivorans]
LeOH11_4325	258.253	9.23787	0.00005	0.000658	-27.9559	Chain C, Aim-1-3mol. Crystal Structure Of The Mobile Metallo-Beta-Lactamase Aim-1 [Pseudomonas Aeruginosa:|Pseudomonas aeruginosa]
LeOH11_1568	30.7633	1.17499	0.00005	0.000658	-26.1818	arabinose efflux permease family protein [Phyllobacterium sp. YR531]
LeOH11_0699	21.9498	0	0.02285	0.098867	-22.9498	NA
LeOH11_0916	428.429	22.8002	0.00005	0.000658	-18.7906	gluconolactonase [Xanthomonas vesicatoria ATCC 35937]
LeOH11_2380	24.1479	2.04207	0.00005	0.000658	-11.8252	NA
LeOH11_2882	8.76051	0.751246	0.01395	0.068479	-11.6613	hypothetical protein [Xanthomonas gardneri]
LeOH11_5154	343.088	29.7761	0.00005	0.000658	-11.5223	NodT family RND efflux system outer membrane lipoprotein [Pseudoxanthomonas spadix BD-a59]
LeOH11_3248	77.8872	6.81158	0.0052	0.03179	-11.4345	NA

LeOH11_1946	10.3073	0	0.02285	0.098867	-11.3073	hypothetical protein [Herbaspirillum seropedicae]
LeOH11_5120	196.366	17.5716	0.00005	0.000658	-11.1752	NA
LeOH11_1462	35.3135	3.25699	0.00005	0.000658	-10.8424	rrf2 family protein transcriptional regulator family protein [Asticcacaulis biprosthecum C19]
LeOH11_5119	172.338	15.9627	0.00005	0.000658	-10.7963	NA
LeOH11_1416	693.607	68.847	0.00005	0.000658	-10.0746	Cyanide-insensitive ubiquinol oxidase, subunit I [Xanthomonas translucens]
LeOH11_5118	363.002	37.3826	0.00005	0.000658	-9.71045	membrane transporter [Pandoraea sp. SD6-2]
LeOH11_5153	515.8	56.2583	0.00005	0.000658	-9.16842	MarR family transcriptional regulator [Pseudoxanthomonas spadix]
LeOH11_4323	35.5703	3.91752	0.00005	0.000658	-9.0798	Glyoxalase/bleomycin resistance protein/dioxygenase [Pseudoxanthomonas suwonensis]
LeOH11_0863	44.5046	4.97326	0.01825	0.084066	-8.94878	NA
LeOH11_5122	7.92608	0	0.00135	0.011151	-8.92608	NA
LeOH11_5117	1844.46	207.467	0.0003	0.003233	-8.89038	Ox4 [Lysobacter enzymogenes]
LeOH11_5155	312.718	36.3186	0.00005	0.000658	-8.61041	multidrug transporter [Pseudoxanthomonas sp. GW2]
LeOH11_1415	720.469	86.2606	0.00005	0.000658	-8.35224	putative cytochrome bd ubiquinol oxidase, subunit II protein [Xanthomonas albilineans GPE PC73]

LeOH11_5116	1640.66	204.672	0.00005	0.000658	-8.01605	Ox3 [Lysobacter enzymogenes]
LeOH11_4533	145.379	19.1608	0.00005	0.000658	-7.58731	hypothetical protein [Amycolatopsis sp. ATCC 39116]
LeOH11_2469	6.04354	0	0.02285	0.098867	-7.04354	NA
LeOH11_0459	16.9317	2.42053	0.00005	0.000658	-6.99504	hypothetical protein [Oxalobacteraceae bacterium JGI 0001004-K23]
LeOH11_3708	242.077	34.9242	0.00025	0.002745	-6.9315	NA
LeOH11_2388	5.5681	0.819946	0.00025	0.002745	-6.79081	NA
LeOH11_1464	21.0654	3.14511	0.00005	0.000658	-6.69783	phosphate-selective porin O and P [Pseudoxanthomonas suwonensis]
LeOH11_3161	26.3546	4.00271	0.0011	0.009582	-6.58419	hypothetical protein [Rudaea cellulosilytica]
LeOH11_5121	16.8363	2.59107	0.00005	0.000658	-6.49782	NA
LeOH11_4412	5.48565	0	0.00135	0.011151	-6.48565	NA
LeOH11_1384	57.6537	8.92206	0.00005	0.000658	-6.46193	conserved hypothetical protein [Xanthomonas albilineans]
LeOH11_3807	14.8456	2.34598	0.00005	0.000658	-6.3281	putative aminoglycoside phosphotransferase [Cystobacter fuscus]
LeOH11_0623	186.272	30.3073	0.00005	0.000658	-6.14611	hypothetical protein [Dyella ginsengisoli]
LeOH11_3790	59.3415	9.85457	0.00125	0.010687	-6.02172	NA
LeOH11_1048	154.484	25.6886	0.00005	0.000658	-6.01372	membrane protein [Xanthomonas campestris]
LeOH11_1989	164.619	27.4405	0.0051	0.031386	-5.99913	putative exonuclease [Pseudoxanthomonas spadix]
LeOH11_2517	3311.7	582.669	0.00005	0.000658	-5.68367	cytochrome BD oxidase subunit I [Rudaea cellulosilytica]

LeOH11_0534	42.9155	7.59479	0.00105	0.009198	-5.65065	hypothetical protein [Stenotrophomonas maltophilia]
LeOH11_3160	25.8512	4.60153	0.00005	0.000658	-5.61796	sulfite reductase (NADPH) hemoprotein, beta-component [Xanthomonas oryzae]
LeOH11_0093	4.54524	0	0.00135	0.011151	-5.54524	NA
LeOH11_2682	452.936	82.5611	0.00005	0.000658	-5.48607	RND transporter, HAE1/HME family, permease protein [Stenotrophomonas sp. SKA14]
LeOH11_3054	33.0441	6.05477	0.00005	0.000658	-5.45753	cystathionine gamma-synthase [Pseudoxanthomonas spadix]
LeOH11_2364	396.335	75.6907	0.00005	0.000658	-5.23624	NA
LeOH11_1988	345.384	66.2898	0.00005	0.000658	-5.21021	helicase [Stenotrophomonas maltophilia]
LeOH11_0084	25.5904	4.93405	0.02545	0.107802	-5.18649	NA
LeOH11_4243	82.2975	16.1063	0.00005	0.000658	-5.10965	FAD binding protein [Cupriavidus sp. UYPR2.512]
LeOH11_1837	4.05126	0	0.02285	0.098867	-5.05126	NA
LeOH11_2784	28.1357	5.66711	0.00005	0.000658	-4.96474	hypothetical protein [Rhodanobacter spathiphylli]
LeOH11_2498	464.353	93.5423	0.00005	0.000658	-4.9641	NA
LeOH11_1569	21.773	4.42252	0.00005	0.000658	-4.92321	acyltransferase [Xanthomonas sacchari]
LeOH11_2419	8.27339	1.70158	0.00005	0.000658	-4.86218	FAD binding domain protein [Serratia odorifera DSM 4582]
LeOH11_5109	252.007	53.6726	0.00005	0.000658	-4.69526	arginase-like protein [Lysobacter enzymogenes]

LeOH11_2518	2139.78	459.622	0.00005	0.000658	-4.65552	cytochrome d ubiquinol oxidase subunit II [Hydrocarboniphaga effusa AP103]
LeOH11_2515	461.691	99.9539	0.00005	0.000658	-4.61904	NA
LeOH11_1914	26.5632	5.7812	0.00465	0.029357	-4.59476	Agmatinase [Xanthomonas translucens]
LeOH11_1418	796.076	174.982	0.00005	0.000658	-4.54947	coproporphyrinogen III oxidase [Stenotrophomonas maltophilia]
LeOH11_1701	475.649	105.425	0.00005	0.000658	-4.51173	aklaviketone reductase [Xanthomonas axonopodis]
LeOH11_0023	126.521	28.1374	0.00005	0.000658	-4.49654	oxidoreductase [Dyella ginsengisoli]
LeOH11_3707	84.0654	18.754	0.00005	0.000658	-4.48253	NA
LeOH11_2514	378.082	85.2383	0.00005	0.000658	-4.43559	hypothetical protein [Rudaea cellulosilytica]
LeOH11_1385	21.8161	4.95097	0.04025	0.151518	-4.40643	conserved hypothetical protein [Delftia acidovorans]
LeOH11_4478	47.2962	10.8336	0.00005	0.000658	-4.3657	major facilitator transporter [Caulobacter sp. AP07]
LeOH11_4530	6.45159	1.48169	0.00005	0.000658	-4.35421	oxidoreductase, FAD-binding protein [Cystobacter fuscus]
LeOH11_2681	426.109	97.9409	0.00005	0.000658	-4.35067	putative RND/Acr family transmembrane transporter [Stenotrophomonas maltophilia K279a]
LeOH11_1290	135.999	31.4325	0.00005	0.000658	-4.3267	NA
LeOH11_2785	24.4314	5.66675	0.00005	0.000658	-4.31136	hypothetical protein [Dyella ginsengisoli]
LeOH11_5111	682.761	159.892	0.00005	0.000658	-4.27014	ferredoxin reductase-like protein [Lysobacter enzymogenes]

LeOH11_2786	15.096	3.53697	0.00005	0.000658	-4.26806	hypothetical protein [Dyella ginsengisoli]
LeOH11_3163	9.76639	2.30135	0.00005	0.000658	-4.24377	hypothetical protein [Rudaea cellulosilytica]
LeOH11_3024	33.915	8.13082	0.00005	0.000658	-4.17117	MFS transporter [Pseudomonas fuscovaginae]
LeOH11_4406	457.138	110.522	0.0017	0.013218	-4.13617	NA
LeOH11_0670	13.4617	3.26257	0.00005	0.000658	-4.1261	outer membrane receptor for ferrienterochelin and colicins [Xanthomonas perforans 91-118]
LeOH11_4436	64.5522	15.6472	0.00005	0.000658	-4.12548	MCP methyltransferase, CheR-type [Pectobacterium carotovorum subsp. carotovorum PC1]
LeOH11_2516	497.999	123.534	0.00005	0.000658	-4.03127	universal stress protein [Cupriavidus pinatubonensis]
LeOH11_4061	91.9366	22.9629	0.00005	0.000658	-4.0037	NAD(P)H dehydrogenase (quinone) [Pseudoxanthomonas suwonensis 11-1]
LeOH11_3390	1246.65	314.85	0.00005	0.000658	-3.9595	hypothetical protein [Thiobacillus thioparus]
LeOH11_0277	355.311	90.463	0.00005	0.000658	-3.92769	NA
LeOH11_0967	165.865	42.3917	0.0001	0.001256	-3.91268	multidrug transporter [Pseudoxanthomonas sp. GW2]
LeOH11_4642	301.582	77.1153	0.00005	0.000658	-3.91079	Fosmidomycin resistance protein [Rhodanobacter sp. 2APBS1]
LeOH11_0458	224.358	57.8341	0.00005	0.000658	-3.87934	NA
LeOH11_2720	6.15518	1.5884	0.00105	0.009198	-3.87508	NA

LeOH11_3056	15.2872	3.96358	0.0082	0.045948	-3.85692	homoserine dehydrogenase [Stenotrophomonas maltophilia]
LeOH11_4534	4.02923	1.04949	0.04	0.151068	-3.83923	membrane protein [Stenotrophomonas maltophilia]
LeOH11_4119	803.649	210.764	0.00005	0.000658	-3.81303	acetyl-CoA acetyltransferase [Xanthomonas arboricola]
LeOH11_2389	6.93145	1.83299	0.0059	0.035278	-3.7815	NA
LeOH11_4447	18.4117	4.9652	0.00005	0.000658	-3.70815	NA
LeOH11_1702	165.518	44.9547	0.00005	0.000658	-3.68188	NA
LeOH11_2521	795.715	216.882	0.00005	0.000658	-3.66888	hypothetical protein [Herbaspirillum sp. JC206]
LeOH11_1596	1091.4	297.688	0.00005	0.000658	-3.66625	electron transport protein SCO1/SenC [Variovorax paradoxus EPS]
LeOH11_2023	2.66075	0	0.00135	0.011151	-3.66075	NA
LeOH11_1915	63.2676	17.3139	0.00005	0.000658	-3.65415	hypothetical protein [Rhodanobacter sp.]
LeOH11_4477	33.8965	9.35592	0.00005	0.000658	-3.623	sugar kinase, ribokinase [Caulobacter sp. AP07]
LeOH11_4647	23.3571	6.45887	0.00215	0.015967	-3.61628	hypothetical protein [Rhodanobacter sp.]
LeOH11_3211	386.974	108.876	0.00005	0.000658	-3.55426	hypothetical protein [Uliginosibacterium gangwonense]
LeOH11_3053	40.6913	11.5207	0.00005	0.000658	-3.53202	Homoserine O-acetyltransferase [Pseudoxanthomonas suwonensis]
LeOH11_3155	26.5495	7.62445	0.00005	0.000658	-3.48215	cysteine synthase [Dyella japonica]
LeOH11_4438	37.1652	10.6826	0.00005	0.000658	-3.47904	Outer membrane autotransporter barrel [Pseudomonas putida]
LeOH11_3805	4.765	1.36983	0.0035	0.023438	-3.47853	MFS transporter [Burkholderia ubonensis]

LeOH11_4568	53.6719	15.4829	0.00005	0.000658	-3.46653	hypothetical protein [Oxalobacteraceae bacterium AB_14]
LeOH11_4120	812.966	234.564	0.00005	0.000658	-3.46586	3-hydroxyacyl-CoA dehydrogenase [Xanthomonas sp. SHU308]
LeOH11_4446	47.2808	13.6436	0.00225	0.016498	-3.46542	hypothetical protein [Dyella ginsengisoli]
LeOH11_0016	98.9328	28.6495	0.00005	0.000658	-3.45321	NA
LeOH11_3114	5.74513	1.67811	0.01295	0.065229	-3.42357	NA
LeOH11_0540	119.12	34.8175	0.00005	0.000658	-3.42127	NA
LeOH11_1226	531.809	155.747	0.00005	0.000658	-3.41457	membrane protein [Stenotrophomonas maltophilia]
LeOH11_1370	35.2093	10.3273	0.00005	0.000658	-3.40934	hypothetical protein [Dyella japonica]
LeOH11_3061	161.596	47.7315	0.00005	0.000658	-3.38552	putative xanthine deshydrogenase [Cupriavidus taiwanensis LMG 19424]
LeOH11_2381	256.969	76.6659	0.00005	0.000658	-3.3518	hypothetical protein [Dyella japonica]
LeOH11_1747	5.69968	1.71695	0.00015	0.001815	-3.31965	hypothetical protein [Hydrogenophaga sp.]
LeOH11_0968	284.964	85.9646	0.00005	0.000658	-3.3149	membrane protein [Xanthomonas axonopodis]
LeOH11_3247	539.419	163.904	0.00005	0.000658	-3.29107	NA
LeOH11_2420	4.54259	1.38903	0.00135	0.011151	-3.27033	hypothetical protein [Serratia odorifera]
LeOH11_4407	189.056	57.9048	0.00005	0.000658	-3.26495	NA
LeOH11_4479	34.3956	10.5517	0.00005	0.000658	-3.25972	hypothetical protein [Oxalobacteraceae bacterium AB_14]

LeOH11_3791	51.0173	15.9808	0.00005	0.000658	-3.19241	NA
LeOH11_3529	78.666	24.644	0.00005	0.000658	-3.1921	pirin [Xanthomonas arboricola]
LeOH11_1829	210.463	66.1954	0.00005	0.000658	-3.17942	putative oxidoreductase, aryl-alcohol dehydrogenase like protein [Rhodanobacter sp. 115]
LeOH11_4643	101.807	33.0046	0.00005	0.000658	-3.08463	DNA-N1-methyladenine dioxygenase [Burkholderia sp. CCGE1003]
LeOH11_1089	27.9886	9.08751	0.00005	0.000658	-3.0799	membrane protein [Stenotrophomonas maltophilia]
LeOH11_2006	21.8083	7.11323	0.01215	0.062351	-3.06588	conserved hypothetical protein [Xanthomonas campestris]
LeOH11_4830	13.5537	4.45939	0.00435	0.027842	-3.03936	conserved hypothetical protein [Chlorobium limicola]
LeOH11_3681	188.768	63.1875	0.00005	0.000658	-2.98743	thiamine pyrophosphate TPP-binding domain-containing protein [Burkholderia sp. CCGE1001]
LeOH11_1533	110.072	36.9631	0.00005	0.000658	-2.97789	trans-aconitate 2-methyltransferase [Amycolatopsis methanolica]
LeOH11_1639	68.1825	23.2677	0.0001	0.001256	-2.93035	hypothetical protein [Dyella japonica]
LeOH11_4476	135.23	46.4754	0.00005	0.000658	-2.90971	transcriptional regulator/sugar kinase [Caulobacter sp. AP07]
LeOH11_0834	1516.72	521.322	0.00005	0.000658	-2.90937	hypothetical protein [Xanthomonas sacchari]
LeOH11_3792	313.866	107.907	0.00005	0.000658	-2.90867	transposase IS3/IS911 family protein [Rhodanobacter thiooxydans]
LeOH11_4437	335.94	115.554	0.00005	0.000658	-2.90721	NA
LeOH11_4192	32.8505	11.3979	0.0002	0.002288	-2.88215	NA

LeOH11_4475	35.4533	12.4899	0.00005	0.000658	-2.83856	thioredoxin domain protein [Caulobacter sp. AP07]
LeOH11_4121	457.306	161.234	0.00005	0.000658	-2.83629	transcriptional regulator, TetR family [Xanthomonas vesicatoria ATCC 35937]
LeOH11_1124	695.439	246.546	0.00005	0.000658	-2.82073	NA
LeOH11_2719	56.5627	20.0553	0.0001	0.001256	-2.82034	NA
LeOH11_2797	222.239	79.5759	0.00005	0.000658	-2.79279	hypothetical protein [Xanthomonas translucens]
LeOH11_3202	149.844	53.6938	0.00005	0.000658	-2.79071	hypothetical protein [Roseomonas sp. B5]
LeOH11_4433	30.4023	10.9603	0.0002	0.002288	-2.77386	short-chain dehydrogenase/reductase SDR [Paenibacillus alvei A6-6i-x]
LeOH11_2877	19.5916	7.06311	0.00175	0.013538	-2.77379	predicted flavoprotein [Hahella chejuensis KCTC 2396]
LeOH11_3680	228.837	82.5571	0.00005	0.000658	-2.77186	Aldehyde Dehydrogenase [Pseudoxanthomonas suwonensis]
LeOH11_1779	408.027	148.317	0.00005	0.000658	-2.75105	hypothetical protein [Xanthomonas axonopodis]
LeOH11_4565	109.246	39.7896	0.00005	0.000658	-2.74559	hypothetical protein [Dyella japonica]
LeOH11_3403	232.053	84.6621	0.00005	0.000658	-2.74093	NA
LeOH11_5167	869.505	318.296	0.00005	0.000658	-2.73175	NA
LeOH11_4535	38.4706	14.1639	0.00005	0.000658	-2.7161	hypothetical protein [Ralstonia sp.]
LeOH11_0919	176.886	65.1328	0.00005	0.000658	-2.71577	putative redox protein, regulator of disulfide bond formation [Acidovorax sp. CF316]

LeOH11_3156	6.04832	2.22839	0.00025	0.002745	-2.71421	sirohydrochlorin ferrochelatase [Pseudoxanthomonas spadix]
LeOH11_3004	178.713	65.927	0.00005	0.000658	-2.71077	PDZ/DHR/GLGF domain-containing protein [Pseudoxanthomonas suwonensis]
LeOH11_4246	192.978	71.2652	0.00005	0.000658	-2.70789	Esterase EstB [Halomonas sp. A3H3]
LeOH11_3119	1932.15	716.496	0.00005	0.000658	-2.69667	sigma-54 modulation protein [Xanthomonas translucens]
LeOH11_0890	31.5702	11.7079	0.00005	0.000658	-2.69649	nuclease [Xanthomonas campestris]
LeOH11_0913	7.25894	2.70137	0.00005	0.000658	-2.68713	major facilitator superfamily [Burkholderia multivorans CGD1]
LeOH11_0900	12.4649	4.66433	0.00035	0.003695	-2.67239	SAM-dependent methlyltransferase [Oceanibaculum indicum]
LeOH11_2475	163.956	61.3752	0.00005	0.000658	-2.67137	NA
LeOH11_2899	246.734	92.8694	0.00005	0.000658	-2.65678	NA
LeOH11_0982	127.081	47.9268	0.00005	0.000658	-2.65156	NA
LeOH11_1465	98.2122	37.1001	0.00005	0.000658	-2.64722	sulfate ABC transporter, periplasmic sulfate-binding protein [Anaeromyxobacter sp. Fw109-5]
LeOH11_4829	19.9781	7.55488	0.00425	0.027468	-2.6444	NA
LeOH11_2394	28.0638	10.6249	0.00005	0.000658	-2.64132	multidrug transporter [Sphingomonas sp. Mn802worker]
LeOH11_4606	261.17	98.9168	0.00005	0.000658	-2.6403	putative beta-lactamase type II [Lysobacter sp. ATCC 53042]
LeOH11_4640	371.396	141.275	0.00005	0.000658	-2.62889	hydrolase [Burkholderia pyrrocinia]

LeOH11_1172	141.104	53.7553	0.00005	0.000658	-2.62493	polysaccharide deacetylase [Stenotrophomonas maltophilia]
LeOH11_0563	77.3392	29.491	0.0212	0.093741	-2.62247	protein of unknown function DUF477 [Stenotrophomonas maltophilia JV3]
LeOH11_1975	532.496	203.149	0.00005	0.000658	-2.62121	hypothetical protein [Moraxella boevrei]
LeOH11_1469	742.984	286.238	0.00005	0.000658	-2.59569	delta-aminolevulinic acid dehydratase [Pseudoxanthomonas spadix]
LeOH11_4405	56.2214	21.6692	0.00245	0.017712	-2.59453	hypothetical protein [Cystobacter fuscus]
LeOH11_4538	2.75024	1.06334	0.0139	0.068379	-2.58642	hypothetical protein [Xanthomonas gardneri]
LeOH11_1866	52.4749	20.4006	0.00005	0.000658	-2.57222	Major Facilitator Superfamily transporter [Caulobacter sp. AP07]
LeOH11_0256	30.1478	11.7213	0.0013	0.010973	-2.57205	putative membrane protein [Xanthomonas axonopodis|Xanthomonas campestris|Xanthomonas citri]
LeOH11_0232	145.381	56.6452	0.00005	0.000658	-2.56652	hypothetical protein [Stenotrophomonas maltophilia]
LeOH11_3034	812.819	318.797	0.00005	0.000658	-2.54964	hypothetical protein [Rhodanobacter fulvus]
LeOH11_2889	498.182	196.021	0.00005	0.000658	-2.54147	hypothetical protein [Xanthomonas campestris]
LeOH11_4448	37.2424	14.7258	0.0205	0.091696	-2.52906	outer membrane efflux protein [Stenotrophomonas sp. SKA14]
LeOH11_3732	26.9183	10.7226	0.001	0.008929	-2.51043	NA
LeOH11_1830	65.0217	25.9259	0.00005	0.000658	-2.50798	putative permease, DMT superfamily [Herbaspirillum sp. YR522]
LeOH11_3005	37.2545	14.963	0.01075	0.056932	-2.48977	NA

LeOH11_0096	98.686	39.6487	0.00005	0.000658	-2.48901	low temperature requirement A protein LtrA [Cellvibrio sp. BR]
LeOH11_1075	9.19856	3.70233	0.0041	0.026685	-2.48453	CTP: molybdopterin cytidylyltransferase [Stenotrophomonas maltophilia]
LeOH11_2915	292.344	117.687	0.00015	0.001815	-2.48408	hypothetical protein [Pseudoxanthomonas sp. GW2]
LeOH11_1597	633.483	255.031	0.00005	0.000658	-2.48395	hypothetical protein [Acidovorax sp. CF316, PMI14_03899]
LeOH11_3206	110.528	44.5174	0.00005	0.000658	-2.4828	hypothetical protein [Rudaea cellulosilytica]
LeOH11_1102	159.765	64.3544	0.00005	0.000658	-2.48258	L-alanine-DL-glutamate epimerase [Rhodanobacter thiooxydans]
LeOH11_4404	318.106	128.158	0.0065	0.037984	-2.48214	hypothetical protein [Myxococcus fulvus]
LeOH11_1913	79.3883	32.0122	0.0213	0.094004	-2.47994	DegT/DnrJ/EryC1/StrS aminotransferase [Rhodanobacter spathiphylli B39]
LeOH11_5165	268.466	108.427	0.00005	0.000658	-2.47601	DNA repair protein RadA/Sms [Xanthomonas translucens]
LeOH11_3209	389.634	157.462	0.00005	0.000658	-2.47446	membrane protein [Xanthomonas arboricola]
LeOH11_1794	177.466	71.7796	0.00005	0.000658	-2.47237	hypothetical protein [Sphingopyxis sp.]
LeOH11_1574	97.7335	39.609	0.00005	0.000658	-2.46746	unknown [Lysobacter enzymogenes]
LeOH11_1750	476.222	193.215	0.00005	0.000658	-2.46473	NA
LeOH11_4079	59.9438	24.3343	0.00005	0.000658	-2.46335	Non-ribosomal peptide synthetase modules (EC 6. 3. 2. -) [Burkholderia rhizoxinica]

LeOH11_3128	959.179	391.797	0.00005	0.000658	-2.44815	conserved hypothetical protein [Xanthomonas oryzae]
LeOH11_1134	18.4737	7.57252	0.0018	0.013879	-2.43957	flagellar P-ring protein FlgI [Burkholderia pyrrocinia]
LeOH11_1686	926.016	379.856	0.00005	0.000658	-2.43781	NA
LeOH11_2400	692.37	284.149	0.00015	0.001815	-2.43664	Rare lipoprotein A [Cystobacter fuscus]
LeOH11_1079	39.3639	16.1647	0.00005	0.000658	-2.43518	tetracenomycin polyketide synthesis O-methyltransferase TcmP [Streptomyces himastatinicus ATCC 53653]
LeOH11_4449	35.8982	14.7419	0.04045	0.151778	-2.43511	hemolysin secretion protein D [Stenotrophomonas maltophilia]
LeOH11_2473	2166.76	890.769	0.00005	0.000658	-2.43246	NA
LeOH11_4650	76.0505	31.2692	0.0013	0.010973	-2.43212	hypothetical protein [uncultured bacterium, ACD_54C00931G0002]
LeOH11_0613	31.8793	13.1285	0.00005	0.000658	-2.42825	hypothetical protein [Pseudoxanthomonas spadix]
LeOH11_3028	595.963	248.354	0.00005	0.000658	-2.39965	acyl-CoA dehydrogenase [Xanthomonas sp. SHU308]
LeOH11_4605	175.824	73.4445	0.00005	0.000658	-2.39397	malate: quinone oxidoreductase [Lysobacter sp. ATCC 53042]
LeOH11_0559	27.4044	11.4806	0.00005	0.000658	-2.38702	NA
LeOH11_3026	33.5299	14.0563	0.00075	0.007137	-2.3854	hypothetical protein [Dyella japonica]
LeOH11_1870	1569.25	658.427	0.00005	0.000658	-2.38333	streptomycin 3''-phosphotransferase [Achromobacter xylosoxidans C54]
LeOH11_1377	39.5784	16.6887	0.00005	0.000658	-2.37157	hypothetical protein [Herbaspirillum lusitanum]
LeOH11_0566	595.818	251.541	0.00005	0.000658	-2.36867	hypothetical protein [Xanthomonas campestris]

LeOH11_0738	17.1138	7.22994	0.0001	0.001256	-2.36707	NA
LeOH11_1916	631.554	266.893	0.0006	0.005878	-2.36632	phosphopantetheine-binding protein [Rhodanobacter thiooxydans]
LeOH11_1322	70.4849	29.8197	0.00005	0.000658	-2.3637	cation/multidrug efflux pump [Opitutaceae bacterium TAV1]
LeOH11_2609	58.9332	24.9329	0.00005	0.000658	-2.36367	Lysophospholipid transporter / 2-acylglycerophosphoethanolamine acyltransferase [Stenotrophomonas maltophilia]
LeOH11_2306	76.0185	32.2069	0.00005	0.000658	-2.36032	Glutaryl-7-ACA acylase [Stenotrophomonas maltophilia]
LeOH11_5206	33.0957	14.0237	0.00005	0.000658	-2.35998	phosphate-binding protein [Stenotrophomonas maltophilia]
LeOH11_4118	333.496	143.017	0.0001	0.001256	-2.33186	hypothetical protein [Zymomonas mobilis]
LeOH11_1131	16.3882	7.04567	0.00025	0.002745	-2.326	hypothetical protein [Burkholderia pyrrocinia]
LeOH11_4445	170.294	73.27	0.0002	0.002288	-2.3242	RNA polymerase subunit sigma-24 [Stenotrophomonas sp. SKA14]
LeOH11_2399	39.7674	17.1186	0.00025	0.002745	-2.32305	NA
LeOH11_3746	1609.03	694.434	0.00005	0.000658	-2.31704	heat shock protein HtpX [Pseudoxanthomonas suwonensis]
LeOH11_1867	94.3256	40.7473	0.00065	0.006301	-2.31489	secretion protein HlyD family protein [Sphingomonas wittichii]
LeOH11_0969	606.226	262.271	0.00005	0.000658	-2.31145	GCN5-related N-acetyltransferase [Pseudomonas mendocina NK-01]
LeOH11_1099	44.8564	19.437	0.00025	0.002745	-2.30778	TonB-denpendent receptor [Rhodanobacter sp. 116-2]
LeOH11_1304	2190.39	949.79	0.00005	0.000658	-2.30618	hypothetical protein [Pseudomonas geniculata]
LeOH11_4564	26.4644	11.513	0.00105	0.009198	-2.29865	hypothetical protein [Pseudomonas fluorescens]

LeOH11_3888	11.239	4.89141	0.0202	0.090617	-2.2977	NA
LeOH11_2552	3337.2	1453.32	0.00005	0.000658	-2.29626	hypothetical protein [Pseudoxanthomonas sp. GW2]
LeOH11_2446	56.1561	24.4756	0.00005	0.000658	-2.29437	hypothetical protein [Asticcacaulis benevestitus]
LeOH11_3017	86.0211	37.5912	0.00005	0.000658	-2.28833	choline dehydrogenase [Xanthomonas translucens]
LeOH11_1957	24.816	10.8736	0.02175	0.095535	-2.28222	hypothetical protein [Catenibacterium mitsuokai]
LeOH11_3167	1692.87	742.577	0.00005	0.000658	-2.27972	transglycosylase [Psychrobacter lutiphocae]
LeOH11_4411	1420.16	626.762	0.00005	0.000658	-2.26587	hypothetical protein [Stenotrophomonas maltophilia]
LeOH11_1941	42.5464	18.784	0.01945	0.088278	-2.26503	NA
LeOH11_2169	1412.07	623.958	0.00005	0.000658	-2.26309	hypothetical protein [Xanthomonas campestris]
LeOH11_0920	268.547	118.702	0.0001	0.001256	-2.26236	NA
LeOH11_0731	60.852	26.9348	0.0001	0.001256	-2.25923	hypothetical protein [Hymenobacter aerophilus]
LeOH11_1191	368.191	163.12	0.00005	0.000658	-2.25718	hypothetical protein [Dyella japonica]
LeOH11_1192	7.54408	3.3453	0.00015	0.001815	-2.25513	Zn-dependent protease with chaperone function, partial [Xanthomonas perforans]
LeOH11_3033	148.615	65.9016	0.04515	0.164227	-2.2551	NA
LeOH11_0862	188.495	83.6558	0.0003	0.003233	-2.25322	XRE family transcriptional regulator [Acidocella sp. MX-AZ02]
LeOH11_2493	8.64413	3.83853	0.03365	0.133163	-2.25194	hypothetical protein [Xanthomonas gardneri]

LeOH11_1125	204.117	90.6556	0.0001	0.001256	-2.25157	TPR repeat-containing protein [Burkholderia ambifaria]
LeOH11_4187	41.5419	18.4587	0.00265	0.018606	-2.25053	NA
LeOH11_1129	21.8026	9.71047	0.00765	0.043391	-2.24527	hypothetical protein [Burkholderia pyrrocinia]
LeOH11_1133	16.4993	7.37002	0.0338	0.133301	-2.2387	hypothetical protein [Cupriavidus sp. WS]
LeOH11_3971	1089.72	487.159	0.00005	0.000658	-2.23689	putative outer membrane antigen lipoprotein [Pseudoxanthomonas suwonensis 11-1]
LeOH11_5211	19.822	8.89372	0.0014	0.011442	-2.22876	putative phosphate transport system-like protein [Pseudoxanthomonas spadix BD-a59]
LeOH11_4077	35.1141	15.7601	0.0002	0.002288	-2.22804	hypothetical protein [Photorhabdus temperata subsp. temperata M1021, B738_02965]
LeOH11_3002	35.6566	16.04	0.00265	0.018606	-2.22298	hypothetical protein [Xanthomonas translucens]
LeOH11_3292	77.5659	34.9056	0.00015	0.001815	-2.22216	hypothetical protein [Dyella ginsengisoli]
LeOH11_4001	20.9669	9.45842	0.00265	0.018606	-2.21674	phosphotyrosine protein phosphatase [Xanthomonas sp. SHU308]
LeOH11_5209	17.4235	7.86558	0.0157	0.075159	-2.21516	phosphate ABC transporter membrane protein 2, PhoT family [Xanthomonas gardneri ATCC 19865]
LeOH11_4413	76.6623	34.6636	0.00025	0.002745	-2.21161	alanine racemase [Dyella japonica]
LeOH11_3690	49.3553	22.3652	0.00035	0.003695	-2.20679	hypothetical protein [Alcanivorax pacificus]

LeOH11_2675	19.5888	8.90364	0.03325	0.132031	-2.20009	NA
LeOH11_3767	726.841	330.719	0.00095	0.008565	-2.19776	SufE protein probably involved in Fe-S center assembly [Xanthomonas citri subsp. citri Aw12879]
LeOH11_3179	14.4703	6.59194	0.0036	0.023798	-2.19515	NA
LeOH11_1101	49.8971	22.7383	0.0003	0.003233	-2.19441	hypothetical protein [Rhodanobacter thiooxydans]
LeOH11_2390	8.43634	3.84489	0.03355	0.132881	-2.19417	endoribonuclease L-PSP [Sinorhizobium meliloti]
LeOH11_2531	147.408	67.29	0.0001	0.001256	-2.19064	hypothetical protein [Xanthomonas sacchari]
LeOH11_1136	28.9527	13.226	0.0066	0.038374	-2.18907	hypothetical protein [Burkholderia pyrrocinia]
LeOH11_2680	513.188	234.716	0.0002	0.002288	-2.18642	TetR family transcriptional regulator [Pseudoxanthomonas spadix]
LeOH11_4418	855.677	391.499	0.00005	0.000658	-2.18564	Outer membrane protein omp38 [Xanthomonas translucens pv. translucens DSM 18974]
LeOH11_4645	133.841	61.376	0.0005	0.004994	-2.18067	hypothetical protein [Rhodanobacter fulvus]
LeOH11_0209	142.818	65.5541	0.00015	0.001815	-2.17863	hypothetical protein [Xanthomonas albilineans]
LeOH11_2749	4.22772	1.94763	0.0027	0.018872	-2.1707	DNA-directed DNA polymerase [Rhodanobacter sp. 115]
LeOH11_1960	1203.74	556.364	0.0005	0.004994	-2.16358	NA
LeOH11_4037	377.758	174.771	0.0005	0.004994	-2.16145	hypothetical protein [Xanthomonas albilineans]
LeOH11_1104	43.6277	20.1952	0.0001	0.001256	-2.1603	hypothetical protein [Rhodanobacter thiooxydans]

LeOH11_2421	10.3534	4.80373	0.0035	0.023438	-2.15528	Methyltransferase type 12 [Burkholderia ambifaria MEX-5]
LeOH11_3532	130.657	60.922	0.00005	0.000658	-2.14466	pirin-related protein [Xanthomonas campestris]
LeOH11_0798	202.723	94.6827	0.00005	0.000658	-2.14108	Mammalian cell entry related domain protein [Pseudoxanthomonas suwonensis 11-1]
LeOH11_0345	206.253	96.3477	0.0003	0.003233	-2.14072	NA
LeOH11_2363	36.2917	16.9543	0.0004	0.00411	-2.14056	NA
LeOH11_1313	221.754	103.606	0.0002	0.002288	-2.14036	hypothetical protein [Rhizobium grahamii]
LeOH11_0565	118.816	55.6193	0.00475	0.029745	-2.13624	hypothetical protein, partial [Xanthomonas axonopodis]
LeOH11_0636	177.465	83.2858	0.0009	0.008194	-2.1308	NA
LeOH11_1874	78.9458	37.0808	0.01225	0.062725	-2.12902	ATPase [Xanthomonas arboricola]
LeOH11_1917	182.51	85.8872	0.0012	0.010355	-2.125	hypothetical protein [Rudaea cellulosilytica]
LeOH11_0558	49.6482	23.4253	0.0006	0.005878	-2.11943	diacylglycerol kinase [Stenotrophomonas maltophilia]
LeOH11_1371	31.5516	14.903	0.00135	0.011151	-2.11713	hypothetical protein [Rhodanobacter sp.]
LeOH11_0633	21.7232	10.2788	0.00025	0.002745	-2.1134	NA
LeOH11_2116	62.2017	29.4386	0.0002	0.002288	-2.11293	isocitrate lyase [Xanthomonas sacchari]
LeOH11_1053	511.806	242.247	0.0002	0.002288	-2.11274	NA
LeOH11_3641	89.7187	42.4663	0.00005	0.000658	-2.1127	3-hydroxyacyl-CoA dehydrogenase [Xanthomonas sp. SHU199]

LeOH11_2007	26.931	12.7649	0.00145	0.011768	-2.10977	outer membrane usher protein FasD [Xanthomonas campestris]
LeOH11_3341	11.595	5.50339	0.0051	0.031386	-2.10688	TonB-dependent receptor [Pseudomonas geniculata]
LeOH11_1748	27.4386	13.0391	0.00035	0.003695	-2.10433	hypothetical protein [Rudaea cellulosilytica]
LeOH11_4402	329.596	156.944	0.0047	0.029592	-2.10009	hypothetical protein [Chondromyces apiculatus|Myxococcus sp.]
LeOH11_0840	73.369	34.9807	0.00085	0.007815	-2.09741	Acetyltransferase, GNAT family protein [Polymorphum gilvum SL003B-26A1]
LeOH11_0026	185.288	88.3463	0.0023	0.016838	-2.09729	geranylgeranyl pyrophosphate synthase [Pseudoxanthomonas spadix BD-a59]
LeOH11_0236	389.97	186.718	0.0001	0.001256	-2.08855	excinuclease ABC subunit B [Xanthomonas translucens]
LeOH11_1757	151.345	72.5457	0.00065	0.006301	-2.0862	hypothetical protein [Herbaspirillum seropedicae]
LeOH11_4934	12.976	6.22281	0.0004	0.00411	-2.08523	hypothetical protein [Rudaea cellulosilytica]
LeOH11_3312	22.5271	10.8435	0.0047	0.029592	-2.07747	GreA/GreB family elongation factor [Methylococcus capsulatus]
LeOH11_3793	237.541	114.348	0.00205	0.015372	-2.07735	conserved hypothetical protein [Comamonas testosteroni]
LeOH11_3006	59.4554	28.6435	0.02385	0.10281	-2.0757	RNA polymerase, sigma-24 subunit, ECF subfamily [Pseudoxanthomonas suwonensis 11-1]
LeOH11_1065	26.6109	12.8251	0.0007	0.006702	-2.07491	cell envelope biogenesis protein TonB [Stenotrophomonas maltophilia]
LeOH11_1130	23.0851	11.1435	0.00105	0.009198	-2.07162	protein of unknown function DUF1078 domain protein [Burkholderia

						ambifaria]
LeOH11_1581	836.092	403.755	0.00025	0.002745	-2.07079	unknown [Lysobacter enzymogenes]
LeOH11_2600	132.8	64.2792	0.00005	0.000658	-2.06599	hypothetical protein [Pseudoxanthomonas spadix]
LeOH11_4683	81.2402	39.3722	0.0021	0.015696	-2.06339	NA
LeOH11_2958	315.766	153.251	0.0003	0.003233	-2.06045	putative ribosomal protein S6 modification protein [Stenotrophomonas maltophilia]
LeOH11_1579	468.479	227.54	0.00045	0.004573	-2.05889	unknown [Lysobacter enzymogenes]
LeOH11_1718	175.941	85.4717	0.00025	0.002745	-2.05847	DNA recombination protein [Azoarcus sp. KH32C]
LeOH11_3709	10.6309	5.1665	0.00235	0.017177	-2.05766	putative Rhs-family exported protein [Stenotrophomonas maltophilia K279a]
LeOH11_4593	11.7945	5.74679	0.0024	0.017432	-2.05236	putative DNA topoisomerase [Lysobacter sp. ATCC 53042]
LeOH11_1918	806.258	393.427	0.0027	0.018872	-2.04932	aminotransferase class-III [Xanthomonas translucens]
LeOH11_1138	16.1711	7.89702	0.0034	0.022934	-2.04775	flagellar M-ring protein FliF [Burkholderia ambifaria]
LeOH11_3346	262.736	129.292	0.00055	0.005446	-2.03211	spermidine N(1)-acetyltransferase [Providencia stuartii MRSN 2154]
LeOH11_5089	23.9974	11.833	0.0024	0.017432	-2.02801	conserved hypothetical protein [Pseudomonas aeruginosa]
LeOH11_0914	25.8284	12.755	0.03545	0.138395	-2.02496	NA
LeOH11_1140	16.7431	8.27001	0.0024	0.017432	-2.02456	NA

LeOH11_0917	193.917	95.8885	0.0007	0.006702	-2.02232	NA
LeOH11_0572	67.1018	33.4309	0.0062	0.036553	-2.00718	efflux transporter, RND family, MFP subunit [Pseudoxanthomonas suwonensis 11-1]
LeOH11_4011	49.3067	24.6244	0.00255	0.018292	-2.00235	type IV secretion protein Rhs [Stenotrophomonas maltophilia]
LeOH11_4398	2143.51	4287.48	0.00025	0.002745	2.000215	SSU ribosomal protein S6P [Xanthomonas axonopodis|Xanthomonas citri|Xanthomonas perforans]
LeOH11_0407	2039.23	4081.73	0.0002	0.002288	2.001604	50S ribosomal protein L18 [Xanthomonas arboricola]
LeOH11_4981	3.31576	6.64024	0.0374	0.144306	2.00263	NA
LeOH11_2206	303.671	608.822	0.00085	0.007815	2.004874	hypothetical protein [Xanthomonas gardneri]
LeOH11_4879	142.715	286.315	0.00015	0.001815	2.006201	murein transglycosylase [Xanthomonas sp. SHU308]
LeOH11_5068	47.9704	96.2847	0.0004	0.00411	2.007169	iron-sulfur cluster-binding protein [Xanthomonas sacchari]
LeOH11_4586	1.43317	2.88196	0.0419	0.156332	2.010899	hypothetical protein [Acidobacteriaceae bacterium KBS 96]
LeOH11_2183	385.341	775.537	0.0002	0.002288	2.012599	hypothetical protein, partial [Xanthomonas sp. SHU199]
LeOH11_2397	6.2732	12.6361	0.0022	0.016234	2.014299	chromosome replication initiation inhibitor protein [Pseudomonas geniculata]
LeOH11_4936	166.935	336.827	0.00035	0.003695	2.017713	hypothetical protein [Burkholderia gladioli]
LeOH11_5081	178.586	360.975	0.00035	0.003695	2.021295	dihydrodipicolinate synthase [Xanthomonas campestris]

LeOH11_5184	2485.46	5024.99	0.0004	0.00411	2.021755	LSU ribosomal protein L27P [Xanthomonas axonopodis|Xanthomonas citri|Xanthomonas perforans]
LeOH11_4435	91.9032	186.238	0.0017	0.013218	2.026458	hypothetical protein [Xanthomonas translucens]
LeOH11_0020	25.9816	52.6813	0.00315	0.021498	2.027639	NA
LeOH11_5062	102.463	208.368	0.00305	0.020846	2.033593	DNA mismatch repair protein [Xanthomonas fragariae]
LeOH11_0841	2419.9	4922.23	0.0001	0.001256	2.034063	30S ribosomal protein S9 [Dyella ginsengisoli]
LeOH11_5052	51.4808	104.812	0.0008	0.007459	2.035943	hypothetical protein [Rhodanobacter spathiphylli]
LeOH11_0426	1403.32	2862.91	0.01095	0.057596	2.040098	translation elongation factor G [Xanthomonas campestris]
LeOH11_4662	7.61895	15.6464	0.00165	0.013026	2.053616	transcriptional regulator, LysR family protein [Pseudoalteromonas agarivorans]
LeOH11_4175	18.1253	37.2566	0.00095	0.008565	2.055503	hypothetical protein, partial [Stenotrophomonas maltophilia]
LeOH11_3995	1.39111	2.85998	0.00805	0.045382	2.055898	putative competence-related dna-transformation transporter protein [Xanthomonas albilineans]
LeOH11_4885	32.575	67.1409	0.00095	0.008565	2.061117	ArsR family transcriptional regulator [Nocardia farcinica]
LeOH11_0941	14.4069	29.7816	0.0012	0.010355	2.067176	hypothetical protein [Xanthomonas sacchari]
LeOH11_3837	26.2451	54.2779	0.0227	0.098772	2.068116	histidinol-phosphate aminotransferase [Xanthomonas sp. SHU199]
LeOH11_4859	354.603	733.689	0.0002	0.002288	2.069043	flavonol synthase [Xanthomonas sacchari]

LeOH11_1392	22.8627	47.5299	0.00025	0.002745	2.078928	LysR family transcriptional regulator [Cupriavidus sp. WS]
LeOH11_2990	3.65791	7.60763	0.00675	0.039051	2.079775	hypothetical protein [Rhodanobacter thiooxydans LCS2, UUA_01784]
LeOH11_4989	63.6348	132.454	0.00015	0.001815	2.081471	hypothetical protein [Thiothrix disciformis]
LeOH11_1326	74.7219	155.576	0.0001	0.001256	2.082067	hypothetical protein [Methylophilus sp. 42]
LeOH11_2269	20.3817	42.4799	0.0013	0.010973	2.084218	NA
LeOH11_1264	271.653	566.901	0.0004	0.00411	2.086857	2-oxo-acid dehydrogenase E1 subunit, homodimeric type [Pseudoxanthomonas suwonensis 11-1]
LeOH11_1379	30.5023	63.7575	0.00135	0.011151	2.090252	hypothetical protein [Variovorax paradoxus]
LeOH11_5104	71.7249	150.075	0.0008	0.007459	2.09237	putative transcriptional regulator, MarR family
LeOH11_3310	11.1201	23.2787	0.03425	0.134504	2.093389	hypothetical protein [Ralstonia sp. AU12-08, C404_22480]
LeOH11_4986	25.6297	53.7956	0.0004	0.00411	2.098956	hypothetical protein, partial [Xanthomonas sp. SHU199]
LeOH11_5019	55.5763	116.747	0.00045	0.004573	2.100662	NA
LeOH11_4400	1485.18	3120.2	0.00005	0.000658	2.10089	50S ribosomal protein L9 [Dyella japonica]
LeOH11_1734	10.895	22.9374	0.00045	0.004573	2.105314	hypothetical protein [Polaromonas sp.]
LeOH11_3377	58.1047	122.343	0.0008	0.007459	2.105561	NA
LeOH11_4968	37.2096	78.4845	0.0007	0.006702	2.109254	glyoxalase [Kaistia granuli]
LeOH11_4577	8.314	17.5392	0.0011	0.009582	2.109598	short-chain dehydrogenase/reductase SDR [Pseudomonas mendocina]

LeOH11_0398	2686.73	5671.99	0.00005	0.000658	2.111113	50S ribosomal protein L17 [Pseudoxanthomonas spadix]
LeOH11_2747	1.69789	3.58613	0.00915	0.05012	2.11211	multidrug ABC transporter ATP-binding protein [Rhodanobacter sp. 116-2]
LeOH11_1333	4.56554	9.69287	0.00475	0.029745	2.12305	NA
LeOH11_4928	631.12	1343.53	0.00015	0.001815	2.128803	protein of unknown function, DUF1993 family [Pseudomonas chlororaphis O6]
LeOH11_2557	1446.2	3082.88	0.0001	0.001256	2.131711	F0F1 ATP synthase subunit delta [Stenotrophomonas maltophilia]
LeOH11_2855	8.36263	17.8287	0.0006	0.005878	2.131949	Glutathione S-transferase [Cystobacter fuscus]
LeOH11_2873	23.3896	49.8999	0.00185	0.014194	2.133423	NA
LeOH11_5077	237.197	506.107	0.00005	0.000658	2.133699	polynucleotide adenylyltransferase [Pseudoxanthomonas suwonensis]
LeOH11_2741	16.0665	34.3643	0.00195	0.014838	2.138879	putative TonB-dependent siderophore receptor [Herminiimonas arsenicoxydans]
LeOH11_3475	32.3514	69.362	0.00005	0.000658	2.144018	hypothetical protein [Proteus penneri]
LeOH11_5054	265.265	573.075	0.00005	0.000658	2.160387	putative deoxycytidine triphosphate deaminase [Xanthomonas axonopodis|Xanthomonas campestris|Xanthomonas euvesicatoria]
LeOH11_3834	212.422	459.024	0.00105	0.009198	2.160906	hypothetical protein [Pseudoxanthomonas spadix BD-a59, DSC_09950]
LeOH11_0424	4926.53	10687.4	0.00005	0.000658	2.169357	30S ribosomal protein S10 [Stenotrophomonas maltophilia]

LeOH11_5063	108.248	235.067	0.0135	0.06698	2.17156	Conserved hypothetical protein [Xanthomonas campestris]
LeOH11_5145	142.341	309.171	0.0005	0.004994	2.172045	16S rRNA-processing protein RimM [Xanthomonas sp. SHU199]
LeOH11_4854	956.164	2078.99	0.00545	0.033057	2.174303	NADH-ubiquinone oxidoreductase chain B [Stenotrophomonas maltophilia]
LeOH11_5038	573.782	1248.2	0.00065	0.006301	2.175391	cell shape determination protein CcmA [Xanthomonas oryzae]
LeOH11_4892	2.38605	5.20116	0.00055	0.005446	2.17982	hypothetical protein [Rudaea cellulosilytica]
LeOH11_4916	81.3735	178.073	0.00005	0.000658	2.188341	NA
LeOH11_2856	16.6209	36.3777	0.0002	0.002288	2.188672	LysR family transcriptional regulator [Cupriavidus taiwanensis]
LeOH11_1076	3.59278	7.86878	0.00345	0.023204	2.190165	NA
LeOH11_1861	28.7273	63.3186	0.00005	0.000658	2.204126	transcriptional regulator [Xanthomonas sp. SHU308]
LeOH11_4245	15.2719	33.9707	0.00085	0.007815	2.224393	PREDICTED: uncharacterized protein LOC101459548 [Ceratitis capitata]
LeOH11_5078	617.879	1374.56	0.00005	0.000658	2.224643	ferredoxin [Pseudoxanthomonas spadix]
LeOH11_3541	89.7858	199.882	0.00005	0.000658	2.226209	cytochrome C551 [Pseudoxanthomonas sp. GW2]
LeOH11_3480	4.90308	10.9174	0.0017	0.013218	2.226641	conserved hypothetical protein [Xanthomonas campestris]
LeOH11_0417	1420.9	3164.94	0.0008	0.007459	2.227419	SSU ribosomal protein S3P [Xanthomonas perforans 91-118]
LeOH11_2403	1.12804	2.51389	0.01535	0.073712	2.228547	Beta-lactamase [Chondromyces apiculatus|Myxococcus sp.]

LeOH11_2894	25.3705	56.6046	0.00005	0.000658	2.231119	Fis family transcriptional regulator [Xanthomonas sp. SHU308]
LeOH11_3441	1.38619	3.09973	0.015	0.072331	2.236151	hypothetical protein [Rudaea cellulosilytica]
LeOH11_5042	61.6738	138.003	0.0007	0.006702	2.237628	hypothetical protein [Xanthomonas vesicatoria]
LeOH11_4839	213.846	478.862	0.00025	0.002745	2.239284	NA
LeOH11_0222	136.074	305.313	0.0002	0.002288	2.243728	NA
LeOH11_4991	33.1508	74.5269	0.00015	0.001815	2.248118	NA
LeOH11_0419	3220.25	7267.21	0.00005	0.000658	2.256722	30S ribosomal protein S19 [Xanthomonas sacchari]
LeOH11_2066	4289.8	9693.97	0.00005	0.000658	2.259772	putative 50S ribosomal protein L28 [Stenotrophomonas maltophilia]
LeOH11_2202	6.19573	14.0175	0.0008	0.007459	2.262445	hypothetical protein [Rudaea cellulosilytica]
LeOH11_2558	730.952	1654.74	0.00055	0.005446	2.263815	putative ATP synthase alpha chain [Stenotrophomonas maltophilia]
LeOH11_2556	512.316	1165.6	0.00005	0.000658	2.275158	F0F1 ATP synthase subunit B [Stenotrophomonas maltophilia]
LeOH11_4862	280.958	641.355	0.00005	0.000658	2.282743	acetyl-CoA carboxylase, carboxyl transferase, beta subunit [Pseudoxanthomonas suwonensis 11-1]
LeOH11_2555	1644.27	3754.23	0.00005	0.000658	2.28322	F0F1 ATP synthase subunit C [Dyella ginsengisoli]
LeOH11_4849	515.664	1178.02	0.0444	0.162757	2.284472	NADH dehydrogenase [Pseudoxanthomonas sp. GW2]
LeOH11_4952	118.183	270.26	0.00005	0.000658	2.286793	chitinase B [Burkholderia gladioli]
LeOH11_4572	2.37661	5.43792	0.0013	0.010973	2.288099	lanthionine synthetase C-like protein [Lysobacter sp. ATCC 53042]

LeOH11_5034	34.267	78.5618	0.00015	0.001815	2.292637	molecular chaperone [Rhodanobacter sp. 2APBS1]
LeOH11_3905	19.629	45.03	0.00005	0.000658	2.294055	MFS transporter [Serratia marcescens]
LeOH11_4963	33.977	78.2521	0.0003	0.003233	2.30309	hypothetical protein [Xanthomonas sp. SHU308]
LeOH11_5020	30.2692	69.7302	0.00025	0.002745	2.303668	hypothetical protein [Rhizobium freirei]
LeOH11_5098	8746.54	20150.4	0.00005	0.000658	2.303814	carbon storage regulator, CsrA [Pseudoxanthomonas suwonensis]
LeOH11_0811	37.7852	87.077	0.00005	0.000658	2.304527	hypothetical protein [Stenotrophomonas maltophilia]
LeOH11_4858	6.53851	15.0912	0.00005	0.000658	2.308049	hypothetical protein [Shewanella denitrificans]
LeOH11_5029	553.742	1279.76	0.00005	0.000658	2.311112	acetyl-CoA acetyltransferase [Rudaea cellulosilytica]
LeOH11_5240	1843.85	4265.08	0.00005	0.000658	2.313138	30S ribosomal protein S2 [Xanthomonas campestris]
LeOH11_3835	63.4434	146.776	0.0036	0.023798	2.313495	ATP phosphoribosyltransferase [Xanthomonas translucens]
LeOH11_5074	16.2039	37.5441	0.0004	0.00411	2.316979	2-amino-4-hydroxy-6-hydroxymethyldihydropteridine pyrophosphokinase [Pseudoxanthomonas sp. GW2]
LeOH11_0418	2247.81	5222.13	0.00005	0.000658	2.323208	probable 50s ribosomal protein l22 [Xanthomonas albilineans|Xanthomonas axonopodis|Xanthomonas campestris|Xanthomonas fuscans]
LeOH11_3321	77.8643	180.968	0.00005	0.000658	2.324146	hypothetical protein [Massilia timonae]
LeOH11_0420	1370.88	3193.35	0.00035	0.003695	2.329416	50S ribosomal protein L2 [Pseudoxanthomonas suwonensis]

LeOH11_4297	149.663	348.762	0.0182	0.083919	2.330315	NA
LeOH11_4925	147.994	345.269	0.00005	0.000658	2.332993	hypothetical protein [Xanthomonas vesicatoria]
LeOH11_4851	385.925	900.667	0.00005	0.000658	2.333788	NADH dehydrogenase subunit D [Pseudoxanthomonas sp. GW2]
LeOH11_0422	2536.79	5922.61	0.00005	0.000658	2.334687	50S ribosomal protein L4 [Pseudoxanthomonas sp. GW2]
LeOH11_5026	208.883	487.796	0.00005	0.000658	2.335259	acyl-CoA dehydrogenase domain-containing protein [Pseudoxanthomonas suwonensis 11-1]
LeOH11_4873	58.376	136.432	0.0065	0.037984	2.337125	Uncharacterized protein SCO1 [Xanthomonas citri subsp. citri Aw12879]
LeOH11_5003	73.3662	171.701	0.0001	0.001256	2.340328	putative methyltransferase [Rhodanobacter sp. 2APBS1]
LeOH11_4965	8.17317	19.1402	0.00005	0.000658	2.341833	NA
LeOH11_1335	13.0116	30.48	0.0007	0.006702	2.342525	cellulose-binding protein [Stenotrophomonas maltophilia]
LeOH11_3135	2.79671	6.55235	0.0004	0.00411	2.342878	NA
LeOH11_2346	113.023	264.889	0.00005	0.000658	2.343673	Hcp family protein [Stenotrophomonas maltophilia]
LeOH11_3904	12.0807	28.3597	0.00005	0.000658	2.347521	hypothetical protein [Janthinobacterium sp. CG3]
LeOH11_3836	33.0593	77.8855	0.0071	0.040821	2.355933	bifunctional histidinal dehydrogenase/ histidinol dehydrogenase [Xanthomonas fuscans]
LeOH11_1672	113.684	268.416	0.00005	0.000658	2.361071	MFS transporter [Stenotrophomonas maltophilia]
LeOH11_5018	18.4394	43.5905	0.0001	0.001256	2.363987	NA

LeOH11_0356	2.56175	6.06146	0.0003	0.003233	2.36614	multidrug ABC transporter ATP-binding protein [Rhodanobacter sp. 116-2]
LeOH11_5032	112.294	266.879	0.00005	0.000658	2.37661	methylcrotonoyl-CoA carboxylase [Pseudoxanthomonas suwonensis]
LeOH11_4641	19.3927	46.2346	0.00005	0.000658	2.384124	hypothetical protein [Rudaea cellulosilytica]
LeOH11_5065	41.9406	100.309	0.0002	0.002288	2.391692	N-acetylmuramoyl-L-alanine amidase [Stenotrophomonas maltophilia]
LeOH11_4850	489.625	1178.21	0.00005	0.000658	2.406352	NADH-quinone oxidoreductase, E subunit [Pseudoxanthomonas suwonensis 11-1]
LeOH11_5047	215.674	519.004	0.0002	0.002288	2.406428	putative membrane protein [Xanthomonas translucens]
LeOH11_5009	37.9724	91.4264	0.00005	0.000658	2.407707	hypothetical protein [Acidobacteriaceae bacterium KBS 89]
LeOH11_3252	131.807	317.473	0.0161	0.076599	2.40862	NA
LeOH11_4883	149.689	361.37	0.00005	0.000658	2.414139	transcription elongation factor GreB [Pseudoxanthomonas suwonensis]
LeOH11_2216	283.889	685.986	0.00005	0.000658	2.416388	hypothetical protein [Massilia niastensis]
LeOH11_2595	131.823	318.949	0.0028	0.019453	2.419525	bacterioferritin [Xanthomonas sacchari]
LeOH11_5015	15.421	37.3867	0.00005	0.000658	2.424402	lytic endopeptidase preproenzyme [Lysobacter sp. XL1]
LeOH11_5231	330.786	805.706	0.00005	0.000658	2.435732	asparagine synthase (glutamine-hydrolysing) [Xanthomonas translucens]
LeOH11_4825	12.5687	30.6247	0.00005	0.000658	2.436585	transcriptional regulator, LysR family [Azotobacter vinelandii]

LeOH11_4960	5.60712	13.7057	0.00215	0.015967	2.444339	HAD-superfamily, subfamily IB hydrolase [Rhodanobacter sp. 115]
LeOH11_3439	8.38134	20.5018	0.0013	0.010973	2.446124	beta-lactamase [Caulobacter sp. K31]
LeOH11_5017	207.14	509.403	0.00005	0.000658	2.459221	hypothetical protein [Rudaea cellulosilytica]
LeOH11_1738	3.60474	8.87562	0.00005	0.000658	2.462208	NA
LeOH11_4864	241.75	595.812	0.00005	0.000658	2.464579	tryptophan synthase subunit beta [Stenotrophomonas maltophilia]
LeOH11_2592	1018.66	2512.24	0.00005	0.000658	2.46622	hypothetical protein [Pseudoxanthomonas sp. GW2]
LeOH11_3671	250.021	617.193	0.00385	0.02527	2.468565	alpha-lytic protease [Lysobacter enzymogenes]
LeOH11_4872	282.407	697.736	0.00005	0.000658	2.470675	protein-(glutamine-N5) methyltransferase, 50S ribosomal protein L3-specific [Pseudoxanthomonas suwonensis 11-1]
LeOH11_4970	45.0314	112.064	0.00005	0.000658	2.488575	LysR family transcriptional regulator [Rhizobium leguminosarum]
LeOH11_0246	294.726	733.611	0.0004	0.00411	2.489129	putative tonb-dependent outer membrane receptor oar-like protein [Xanthomonas albilineans]
LeOH11_2325	2.28785	5.7006	0.00105	0.009198	2.491684	NA
LeOH11_5040	44.5637	111.442	0.00005	0.000658	2.500735	NA
LeOH11_5031	106.554	267.095	0.00005	0.000658	2.506663	NA
LeOH11_4852	501.435	1257.67	0.00475	0.029745	2.508142	NADH dehydrogenase subunit C [Pseudoxanthomonas sp. GW2]
LeOH11_3322	22.2164	55.9831	0.00005	0.000658	2.5199	TfoX domain-containing protein [Collimonas fungivorans]

LeOH11_0885	146.866	371.193	0.00005	0.000658	2.527426	amino acid/peptide transporter (Peptide: H+ symporter) [Stenotrophomonas maltophilia Ab55555]
LeOH11_4996	212.092	537.162	0.00005	0.000658	2.532684	peptidase [Dyella ginsengisoli]
LeOH11_2225	974.501	2476.71	0.00035	0.003695	2.541516	TonB-denpendent receptor [Pseudomonas geniculata]
LeOH11_4947	1.50117	3.82191	0.0012	0.010355	2.545954	hypothetical protein [Xanthomonas campestris]
LeOH11_2898	28.8983	73.6167	0.00005	0.000658	2.547441	RNA 3'-terminal-phosphate cyclase [Xanthomonas sacchari]
LeOH11_5093	147.886	377.5	0.0002	0.002288	2.552642	NA
LeOH11_4875	1.59997	4.0894	0.00025	0.002745	2.555923	putative integral membrane sensor protein
LeOH11_5058	108.21	276.985	0.00005	0.000658	2.559699	methionyl-tRNA ligase [Xanthomonas translucens]
LeOH11_2845	1.21109	3.1064	0.04635	0.167279	2.564962	hypothetical protein CATMIT_01598, partial [Catenibacterium mitsuokai DSM 15897]
LeOH11_2057	5.74376	14.735	0.0045	0.028605	2.565393	NA
LeOH11_1735	1.49811	3.85327	0.03285	0.130667	2.572087	hypothetical protein [Polaromonas naphthalenivorans]
LeOH11_4977	128.075	329.468	0.00005	0.000658	2.572461	hypothetical protein [Variovorax sp.]
LeOH11_4995	70.1725	180.519	0.00005	0.000658	2.572503	hypothetical protein CATMIT_01901, partial [Catenibacterium mitsuokai DSM 15897]
LeOH11_5023	14.9714	38.5406	0.00005	0.000658	2.574282	2OG-Fe(II) oxygenase [Xanthomonas axonopodis]

LeOH11_0688	360.533	932.936	0.00005	0.000658	2.587658	TonB-dependent outer membrane receptor [Xanthomonas axonopodis pv. citrumelo F1]
LeOH11_4895	78.034	202.174	0.00005	0.000658	2.590845	thioesterase [Xanthomonas sacchari]
LeOH11_4953	18.1807	47.3089	0.00005	0.000658	2.60215	NA
LeOH11_0291	19.9966	52.5342	0.0001	0.001256	2.627157	carbon starvation protein A [Xanthomonas axonopodis]
LeOH11_4863	118.451	313.275	0.00005	0.000658	2.644765	tryptophan synthase subunit alpha [Xanthomonas sacchari]
LeOH11_4867	61.7081	163.424	0.00005	0.000658	2.64834	hypothetical protein [Xylella fastidiosa]
LeOH11_5163	10.7314	28.4333	0.01275	0.064643	2.649542	NA
LeOH11_4886	60.6415	160.688	0.00005	0.000658	2.649803	putative activator of Hsp90 ATPase homologue 1-like [Ralstonia solanacearum]
LeOH11_1552	51.6976	137.232	0.00005	0.000658	2.654514	TetR family transcriptional regulator [Myxococcus stipitatus]
LeOH11_4914	21.0311	55.8735	0.00005	0.000658	2.656708	NAD(FAD)-utilizing dehydrogenase [Pseudoxanthomonas sp. GW2]
LeOH11_1554	5.81638	15.4966	0.001	0.008929	2.664303	hypothetical protein [Xanthomonas gardneri]
LeOH11_5059	16.6381	44.3777	0.00015	0.001815	2.667234	ferredoxin [Stenotrophomonas maltophilia]
LeOH11_5043	51.3552	137.357	0.00005	0.000658	2.674646	ferrous iron transport protein B [Pseudoxanthomonas spadix BD-a59]
LeOH11_5039	369.047	987.205	0.00815	0.045778	2.675012	hypothetical protein [Xanthomonas sp. SHU308]
LeOH11_2745	2073.56	5550.11	0.00005	0.000658	2.676609	NA

LeOH11_0214	6.63742	17.8219	0.00025	0.002745	2.685064	membrane protein, putative [Pseudomonas aeruginosa]
LeOH11_3224	17.656	47.8271	0.00005	0.000658	2.70883	peptidase S9 prolyl oligopeptidase active site domain-containing protein [Stenotrophomonas maltophilia]
LeOH11_4695	17.2001	46.6941	0.00265	0.018606	2.714757	NA
LeOH11_4966	3.0769	8.35605	0.00205	0.015372	2.715737	Permease of the drug/metabolite transporter (DMT) superfamily [Cystobacter fuscus]
LeOH11_4911	10.8401	29.453	0.00005	0.000658	2.717041	NA
LeOH11_5016	72.9279	201.876	0.0002	0.002288	2.768159	hypothetical protein [Moritella sp.]
LeOH11_4579	6.29641	17.4476	0.00005	0.000658	2.771039	NA
LeOH11_4169	2.78805	7.75296	0.00045	0.004573	2.780782	citrate transporter [Azoarcus sp. KH32C]
LeOH11_5025	87.2695	243.521	0.00005	0.000658	2.790448	hypothetical protein [Enterococcus moraviensis]
LeOH11_4460	3.74356	10.4583	0.00005	0.000658	2.793678	sulfatase [Myxococcus stipitatus]
LeOH11_5021	177.624	499.22	0.00005	0.000658	2.810544	methylmalonyl-CoA mutase [Dyella ginsengisoli]
LeOH11_2462	488.403	1374.24	0.0039	0.025526	2.813742	methylmalonate-semialdehyde dehydrogenase [Stenotrophomonas maltophilia]
LeOH11_2991	7.56798	21.2977	0.00005	0.000658	2.814186	outer membrane receptor protein, mostly Fe transport [Stenotrophomonas sp. SKA14]

LeOH11_4856	199.519	561.92	0.00005	0.000658	2.816373	putative triosephosphate isomerase [Stenotrophomonas maltophilia]
LeOH11_1758	3.9841	11.2486	0.00005	0.000658	2.823373	hypothetical protein [Ralstonia sp.]
LeOH11_5053	424.476	1211.19	0.00005	0.000658	2.853377	hypothetical protein [Xanthomonas sp. SHU199]
LeOH11_5055	81.462	232.587	0.00005	0.000658	2.855159	sodium: proton antiporter [Xanthomonas oryzae]
LeOH11_2285	18.3857	53.127	0.00005	0.000658	2.889583	NA
LeOH11_4877	5.42665	15.6846	0.0005	0.004994	2.890291	NA
LeOH11_1556	11.4142	33.142	0.00005	0.000658	2.903576	sulfonate ABC transporter permease [Xanthomonas sacchari]
LeOH11_5006	25.3041	73.539	0.00005	0.000658	2.906209	conserved hypothetical protein [Methylobacterium extorquens]
LeOH11_4987	3.91685	11.574	0.00005	0.000658	2.954926	PHB depolymerase PhaZ7 precursor [Paucimonas lemoignei]
LeOH11_4949	3.8077	11.2629	0.00005	0.000658	2.957927	hypothetical protein with peptidoglycan-binding domain [Xanthomonas campestris]
LeOH11_5142	3375.48	10056	0.00005	0.000658	2.979132	50S ribosomal protein L19 [Xanthomonas sacchari]
LeOH11_2659	9118.03	27553	0.00125	0.010687	3.021815	GroEL [Acinetobacter baumannii]
LeOH11_5160	42.0696	129.402	0.00035	0.003695	3.075903	NA
LeOH11_5230	85.6175	263.453	0.00005	0.000658	3.077093	NA
LeOH11_1275	11.9831	37.0717	0.00645	0.037883	3.093665	transcriptional regulator [Stenotrophomonas maltophilia]
LeOH11_4893	43.8652	135.99	0.00005	0.000658	3.10018	NA

LeOH11_3251	81.2647	252.105	0.00005	0.000658	3.102269	D-3-phosphoglycerate dehydrogenase [Geobacillus sp.|Geobacillus thermoglucosidans]
LeOH11_1859	8.66022	27.1505	0.00005	0.000658	3.135082	TonB-dependent receptor protein [Rhodanobacter sp. 116-2]
LeOH11_5064	22.4242	70.6916	0.00005	0.000658	3.152469	NA
LeOH11_2658	7466.97	23616.3	0.00005	0.000658	3.162769	molecular chaperone GroES [Stenotrophomonas maltophilia]
LeOH11_5218	1.58414	5.06422	0.02925	0.120699	3.196826	hypothetical protein [Roseovarius nubinhibens]
LeOH11_0088	40.303	128.857	0.00005	0.000658	3.197206	RNA helicase, partial [Xanthomonas axonopodis]
LeOH11_2179	9.05371	29.128	0.00005	0.000658	3.217245	conserved hypothetical protein [Ralstonia syzygii R24]
LeOH11_2660	10.4293	33.5572	0.00005	0.000658	3.217589	hypothetical protein [Micromonospora sp.]
LeOH11_3177	44.8872	147.192	0.00005	0.000658	3.279153	hypothetical protein [Rudaea cellulosilytica]
LeOH11_1625	272.624	896.987	0.00005	0.000658	3.290198	glucose/galactose transporter [Xanthomonas gardneri ATCC 19865]
LeOH11_4973	1.68977	5.5669	0.00425	0.027468	3.294472	hypothetical protein [Phaeobacter gallaeciensis]
LeOH11_4281	13.7882	45.6487	0.00005	0.000658	3.310708	peptidase M56 BlaR1 [Stenotrophomonas maltophilia]
LeOH11_3253	78.1743	260.619	0.00005	0.000658	3.333819	Phosphoglycerate mutase [Pseudoxanthomonas suwonensis]
LeOH11_0079	9.85513	33.221	0.00005	0.000658	3.370935	hypothetical protein [Xanthomonas sp. SHU199]
LeOH11_4894	181.541	631.165	0.00005	0.000658	3.476708	hypothetical protein [Nematostella vectensis, NEMVEDRAFT_v1g144964]

LeOH11_4823	14.8996	51.9245	0.00005	0.000658	3.484959	transcriptional regulator, LysR family [Agrobacterium fabrum str. C58]
LeOH11_2137	27.7213	97.2492	0.00005	0.000658	3.508104	LysR family transcriptional regulator [Pseudoxanthomonas spadix]
LeOH11_1179	63.004	221.053	0.00005	0.000658	3.508555	conserved hypothetical protein [Xanthomonas campestris]
LeOH11_4855	305.725	1073.13	0.00005	0.000658	3.510115	preprotein translocase subunit SecG [Pseudoxanthomonas sp. GW2]
LeOH11_5045	88.7191	312.502	0.00005	0.000658	3.522376	enoyl-CoA hydratase/isomerase [Pseudoxanthomonas suwonensis]
LeOH11_5030	2803.88	9939.76	0.00005	0.000658	3.545002	NA
LeOH11_3443	0.922023	3.28062	0.01	0.053884	3.558067	hypothetical protein [Rudaea cellulosilytica]
LeOH11_4896	8.49133	30.5274	0.00005	0.000658	3.595126	Acriflavin resistance protein [Azospirillum amazonense Y2]
LeOH11_4999	58.2553	209.778	0.00005	0.000658	3.601011	hypothetical protein [Kordiimonas gwangyangensis]
LeOH11_4280	50.8359	186.24	0.00005	0.000658	3.663553	Uncharacterized 16. 5 kDa protein in ptsI 3'region [Stenotrophomonas maltophilia]
LeOH11_4876	6.07513	22.5892	0.00005	0.000658	3.718307	hypothetical protein [Xanthomonas vesicatoria]
LeOH11_4920	1.26555	4.70996	0.00005	0.000658	3.72167	putative autotransporter protein, putative Ig domain-containing protein [Rhodanobacter sp. 2APBS1]
LeOH11_3223	123.53	472.887	0.00005	0.000658	3.828115	TonB-dependent receptor [Stenotrophomonas maltophilia]
LeOH11_4998	8.76482	33.8282	0.00005	0.000658	3.859543	membrane protein [Rhizobium sp. BR816]
LeOH11_1277	5.58654	21.6461	0.00005	0.000658	3.874688	citrate transporter [Stenotrophomonas maltophilia]

LeOH11_1759	1.57958	6.29917	0.0097	0.052634	3.987877	NA
LeOH11_5159	16.3896	65.6048	0.0028	0.019453	4.002831	NA
LeOH11_1609	12.5179	50.2319	0.00005	0.000658	4.012806	nitrogen regulatory protein P-II 2 [Rhodanobacter sp. 116-2]
LeOH11_2897	32.5324	131.095	0.00005	0.000658	4.029675	NA
LeOH11_4945	1.17442	4.90217	0.00185	0.014194	4.17412	hypothetical protein [Xanthomonas sacchari]
LeOH11_4955	15.5081	65.4311	0.00005	0.000658	4.219156	NA
LeOH11_5229	11.9269	50.7997	0.00005	0.000658	4.259254	NA
LeOH11_4170	5.72478	25.0118	0.00005	0.000658	4.369041	TonB-dependent receptor [Stenotrophomonas sp. SKA14]
LeOH11_3058	14.1977	62.1043	0.0079	0.04459	4.374251	NA
LeOH11_4279	4.84	21.4122	0.00005	0.000658	4.424008	beta-lactamase [Stenotrophomonas maltophilia]
LeOH11_4898	16.8684	77.672	0.00005	0.000658	4.604586	diaminobutyrate--2-oxoglutarate aminotransferase [Photorhabdus luminescens]
LeOH11_1177	43.0718	199.426	0.00005	0.000658	4.630083	hypothetical protein [Sphingobium sp.]
LeOH11_1629	12.0852	57.5373	0.00005	0.000658	4.760972	beta-hexosaminidase [Azospirillum amazonense]
LeOH11_5046	35.4523	171.787	0.00005	0.000658	4.845581	NA
LeOH11_5170	65.9903	323.253	0.00005	0.000658	4.898493	hypothetical protein [Cellvibrio sp. BR, O59_002925]
LeOH11_1764	1.0094	4.9692	0.0125	0.063724	4.922925	hypothetical protein [Ralstonia sp.]

LeOH11_4897	4.19278	20.686	0.00005	0.000658	4.933719	NA
LeOH11_4899	8.90372	43.93	0.0009	0.008194	4.933893	hypothetical protein [Teredinibacter turnerae]
LeOH11_4923	7.50624	37.1547	0.00005	0.000658	4.949842	microcystin-dependent protein [Phyllobacterium sp. YR531]
LeOH11_2178	25.5206	129.533	0.00005	0.000658	5.075625	hypothetical protein [Pseudanabaena sp. PCC 6802]
LeOH11_1551	7.59792	38.5685	0.00005	0.000658	5.076192	hypothetical protein [Chelatococcus sp. GW1]
LeOH11_1176	51.514	263.17	0.00005	0.000658	5.108708	hypothetical protein [Dyella ginsengisoli]
LeOH11_2348	2.35289	12.2865	0.0008	0.007459	5.221876	type VI secretion protein [Stenotrophomonas maltophilia]
LeOH11_3221	74.1927	399.144	0.00005	0.000658	5.379828	translation elongation factor G [Stenotrophomonas maltophilia]
LeOH11_1608	9.92436	53.9874	0.00005	0.000658	5.439887	ammonia channel protein [Dyella ginsengisoli]
LeOH11_1612	789.966	4376.72	0.00005	0.000658	5.54039	probable glutamate--ammonia ligase protein [Xanthomonas albilineans GPE PC73]
LeOH11_3059	23.6209	131.673	0.00005	0.000658	5.574428	chitinase A [Lysobacter enzymogenes]
LeOH11_4307	36.3784	206.402	0.0002	0.002288	5.673751	NA
LeOH11_4908	2.90462	16.6426	0.00005	0.000658	5.7297	hypothetical protein [Janthinobacterium sp. CG3]
LeOH11_1610	12.142	69.6135	0.00005	0.000658	5.733281	hypothetical protein [Dyella ginsengisoli]
LeOH11_1611	84.6879	489.183	0.00005	0.000658	5.776303	Histone acetyltransferase HPA2/related acetyltransferase [Hahella chejuensis KCTC 2396]

LeOH11_1020	52.7045	333.779	0.00005	0.000658	6.333027	probable short-chain dehydrogenase [Sorangium cellulosum So ce56]
LeOH11_4924	4.42343	28.2385	0.00005	0.000658	6.383847	acetyltransferase [Collimonas fungivorans]
LeOH11_4905	20.4822	131.073	0.00005	0.000658	6.399361	hypothetical protein [Dickeya zeae]
LeOH11_4904	35.3319	226.971	0.00005	0.000658	6.423968	glycine C-acetyltransferase [Dickeya dadantii Ech586]
LeOH11_4992	2.23838	14.4489	0.00005	0.000658	6.45507	monophenol monooxygenase [Stenotrophomonas maltophilia JV3]
LeOH11_0674	31.7703	207.874	0.00005	0.000658	6.543029	hypothetical protein [Singularimonas variicoloris]
LeOH11_1276	3.24546	21.252	0.00005	0.000658	6.548224	Porin P [Xanthomonas translucens]
LeOH11_2052	3.02954	20.7747	0.00005	0.000658	6.857378	major facilitator transporter [Chelatococcus sp. GW1]
LeOH11_4575	0.828233	5.87431	0.00005	0.000658	7.092581	Phosphate-specific outer membrane porin OprP, Pyrophosphate-specific outer membrane porin OprO [Stenotrophomonas maltophilia]
LeOH11_4979	11.4552	84.4585	0.00005	0.000658	7.37294	Lipase [Xanthomonas translucens]
LeOH11_4903	46.2442	342.23	0.00005	0.000658	7.400496	hypothetical protein [Teredinibacter turnerae]
LeOH11_4574	1.02784	7.73204	0.00015	0.001815	7.522611	Endo-1, 4-beta-xylanase B B [Stenotrophomonas maltophilia]
LeOH11_0369	6.48854	48.8565	0.00005	0.000658	7.52966	MFS transporter [Pseudoxanthomonas sp. GW2]
LeOH11_4922	4.4807	35.136	0.00005	0.000658	7.841632	microcystin-dependent protein [Phyllobacterium sp. YR531]
LeOH11_4921	9.20139	74.9445	0.00005	0.000658	8.144911	microcystin-dependent protein [Phyllobacterium sp. YR531]
LeOH11_3037	20.4057	168.765	0.00005	0.000658	8.270483	TonB-dependent receptor [Xanthomonas oryzae]

LeOH11_1760	1.96364	16.5147	0.0002	0.002288	8.410248	peptidase C39 [Burkholderia pyrrocinia]
LeOH11_0371	11.1573	94.1202	0.00005	0.000658	8.435751	c4-dicarboxylate transporter dcta [Hydrocarboniphaga effusa AP103]
LeOH11_2054	1.53463	14.2798	0.00005	0.000658	9.305044	hypothetical protein [Pseudoxanthomonas sp. GW2]
LeOH11_1762	2.74959	26.5211	0.00005	0.000658	9.645474	hypothetical protein [Cupriavidus sp. WS]
LeOH11_4306	48.0224	467.461	0.00005	0.000658	9.734228	NAD(P)H dehydrogenase [Pseudomonas aeruginosa]
LeOH11_1627	26.3022	289.602	0.00005	0.000658	11.01056	glucokinase [Xanthomonas fuscans]
LeOH11_0372	5.80129	73.6671	0.00005	0.000658	12.6984	porin [Xanthomonas sacchari]
LeOH11_1628	83.2464	1162.31	0.00005	0.000658	13.96229	TonB-denpendent receptor [Stenotrophomonas maltophilia]
LeOH11_1763	1.10896	18.0349	0.0026	0.018508	16.26289	NA
LeOH11_2569	6.8923	119.348	0.00005	0.000658	17.31614	protein of unknown function DUF214 [Pseudoxanthomonas suwonensis 11-1]
LeOH11_2568	1.0332	19.0735	0.00005	0.000658	18.46061	X-Pro dipeptidyl-peptidase [Rhodanobacter thiooxydans LCS2]
LeOH11_2570	6.91685	145.601	0.00005	0.000658	21.05019	ABC transporter ATP-binding protein [Xanthomonas sacchari]
LeOH11_3663	0	24.6423	0.00005	0.000658	25.6423	Zn-dependent protease with chaperone function [Acidovorax sp. MR-S7]
LeOH11_2573	1.62645	42.3321	0.00005	0.000658	26.0273	ABC transporter permease [Xanthomonas sacchari]
LeOH11_2572	9.55172	256.383	0.00285	0.019682	26.84155	NA
LeOH11_3654	0.107149	29.7286	0.0026	0.018508	27.75471	NA

LeOH11_2571	4.42808	157.243	0.00005	0.000658	35.51042	phosphonate ABC transporter ATP-binding protein [Xanthomonas campestris]
LeOH11_5108	9.42651	536.246	0.00005	0.000658	56.88701	TonB-dependent outer membrane receptor precursor [Xanthomonas campestris]
LeOH11_3169	22.9961	1683.48	0.00005	0.000658	73.2072	putative comea-related dna uptake protein [Xanthomonas albilineans]
LeOH11_3661	0.32877	128.865	0.04035	0.151648	97.73324	5'-methylthioadenosine phosphorylase [Stenotrophomonas maltophilia]
LeOH11_3656	0.390506	183.52	0.0026	0.018508	132.6999	23S rRNA 5-methyluridine methyltransferase [Xanthomonas campestris]

Table S2 Distribution of the HSAF biosynthetic gene operon in selected *Lysobacter* members

***Lysobacter* Species and strains**	**Presnce (+) or absence (-) of the HSAF biosynthetic gene operon**
Lysobacter capsici 55	+
Lysobacter capsici AZ78	+
Lysobacter gummosus OH17	+
Lysobacter gummosus 3.2.11	+
Lysobacter antibioticus 13-6	-
Lysobacter antibioticus OH13	-
Lysobacter antibioticus ATCC29479	-
Lysobacter antibioticus HS124	+
Lysobacter antibioticus 76	-
Lysobacter enzymogenes ATCC29487	-
Lysobacter enzymogenes C3	+
Lysobacter enzymogenes OH11	+
*Lysobacter d*aejeonensis GH1-9	-
Lysobacter brunescens OH21	-

Table S3 Bacterial strains and plasmids used in this study

Strain or plasmid	Characteristics[a]	Source
**Strains**		
Lysobacter enzymogenes OH11	Wild type, Km^R	Lab stock
Xanthomonas oryzae pv. *oryzae*	Wild type, Rif^R	Lab stock
Valsa pyri SXYL134	Wild type	Lab stock
XL1-Blue MRF' Kan	Δ(*mcrA*)183Δ(mcrCB-hsdSMR-mrr)173,*endA1,supE44,thi-1,recA1 gyrA96,relA1*,lac, [F' *proAB laclqZ*ΔM15 Tn5], Km^R	(Qian, et al. 2009; Harris and Merrick 2015)
XL1-Blue(pBX-pTRG)	XL1-Blue harbour empty pBX and pTRG, Tet^R, $Chlo^R$	Lab stock
XL1-Blue(pBX-*lafB*&pTRG-FleQ)	XL1-Blue harbour empty pBX-*lafB* and pTRG-FleQ, Tet^R, $Chlo^R$	This study
WT_{OH11}(*gfp*)	WT_{OH11} harbouring plasmid pBBR-GFP-FLAG, Km^R, Gm^R	This study
WT_{OH11}(FleQ)	WT_{OH11} harbouring plasmid pBBR-$FleQ_{Xoo}$-FLAG, Km^R, Gm^R	This study
WT_{OH11}($FleQ^{\Delta FleQ}$)	WT_{OH11} harbouring plasmid pBBR- $FleQ^{\Delta FleQ}$ -FLAG, Km^R, Gm^R	This study
WT_{OH11}($FleQ^{\Delta HTH}$)	WT_{OH11} harbouring plasmid pBBR- $FleQ^{\Delta HTH}$ -FLAG, Km^R, Gm^R	This study
WT_{OH11}(FliG-GFP)	WT_{OH11} harbouring plasmid pBBR-FliG-*gfp*, Km^R, Gm^R	This study
WT_{OH11}(FliE-GFP)	WT_{OH11} harbouring plasmid pBBR-FliE-*gfp*, Km^R, Gm^R	This study
WT_{OH11}(MotA-GFP)	WT_{OH11} harbouring plasmid pBBR-MotA-*gfp*, Km^R, Gm^R	This study

WT_{OH11}(MotB-GFP)	WT_{OH11} harbouring plasmid pBBR-MotB-*gfp*, Km^R, Gm^R	This study
WT_{OH11}(FlgL-GFP)	WT_{OH11} harbouring plasmid pBBR-FlgL-*gfp*, Km^R, Gm^R	This study
WT_{OH11}(FliD-GFP)	WT_{OH11} harbouring plasmid pBBR-FliD-*gfp*, Km^R, Gm^R	This study
**plasmids**		
pTRG	The plasmid used for protein expression in bacterial one-hybridization assay, Tet^R	(Qian, et al. 2009)
pBXcmT	The plasmid used for promoter in bacterial one-hybridization assay, $Chlo^R$	(Fang, et al. 2014)
pBBR-*gfp*-FLAG	pBBR1-MCS5 carrying *gfp*-FLAG coding region, Gm^R	This study
pBBR-FleQ-FLAG	pBBR1-MCS5 carrying FleQ-FLAG coding region, Gm^R	This study
pBBR-$FleQ^{\Delta FleQ}$-FLAG	pBBR1-MCS5 carrying $FleQ^{\Delta FleQ}$-FLAG coding region, Gm^R	This study
pBBR-$FleQ^{\Delta HTH}$-FLAG	pBBR1-MCS5 carrying $FleQ^{\Delta HTH}$-FLAG coding region, Gm^R	This study
pBBR-FliG-*gfp*	pBBR1-MCS5 carrying FliG-*gfp* coding region, Gm^R	This study
pBBR-FliD-*gfp*	pBBR1-MCS5 carrying FliD-*gfp* coding region, Gm^R	This study
pBBR-MotA-*gfp*	pBBR1-MCS5 carrying MotA-*gfp* coding region, Gm^R	This study
pBBR-MotB-*gfp*	pBBR1-MCS5 carrying MotB-*gfp* coding region, Gm^R	This study
pBBR-FliE-*gfp*	pBBR1-MCS5 carrying FliE-*gfp* coding region, Gm^R	This study
pBBR-FlgL-*gfp*	pBBR1-MCS5 carrying FlgL-*gfp* coding region, Gm^R	This study
pBX-*lafB*	pBX with the promotor of *lafB*, Chl^R	This study

pTRG-FleQ	pTRG with the coding region of FleQ from strain *Xoo*, Tet^R	This study

[a]Km^R, Gm^R Amp^R, Tet^R, $Chlo^R$, kanamycin, gentamicin, ampicillin, tetracycline, chloramphenicol resistance, respectively.

Table S4 PCR primers used in this study

Primers	5'-3' sequencea	Function
FleQ-FLAG-F	CCCAAGCTTGATGAGTGAGTCCCGCATTCT	To generate
FleQ-FLAG-R	CGGGATCCTCACTTATCGTCGTCATCCTTGTAATCGTTGGCCAGCTCGGTCTGCT	pBBR-FleQ-FLAG
FleQ$^{\Delta HTH}$-FLAG-F	CCCAAGCTTGATGAGTGAGTCCCGCATTCT	To generate
FleQ$^{\Delta HTH}$ -FLAG-R	CGGGATCCTCACTTATCGTCGTCATCCTTGTAATCGTGGCCACGCAAATCGATAC	pBBR-Le4230-FLAG
FleQ$^{\Delta FleQ}$ -FLAG-F	CCCAAGCTTGACCGGCAATAGCGAAGCGGTGACCCGCCT	To generate
FleQ$^{\Delta FleQ}$ -FLAG-R	CGGGATCCTCACTTATCGTCGTCATCCTTGTAATCGTTGGCCAGCTCGGTCTGCTCGCG	pBBR-Le4253-FLAG
gfp-F	AGGGAACAAAAGCTGGGTACCGATGAGTAAAGGTGAAGAACT	To generate
gfp-R	AGGAATTCGATATCAAGCTTTCACTTATCGTCGTCATCCTTGTAATCTTTGTAGAGTTCA	pBBR-*gfp*
FliD-F	AGGGAACAAAAGCTGGGTACCGATGGCATCGGTGATCAGCACTTCC	To generate
FliD--R	GAACAGTTCTTCACCTTTACTCATGCCTGTCAGCAGGCCGCTGAGCGA	pBBR-FliD-gfp
FlgL-F	AGGGAACAAAAGCTGGGTACCGATGACCGACCGTATCTCTACCAGC	To generate
FlgL-R	GAACAGTTCTTCACCTTTACTCATGCGGATCAGGTTGAACAACGACGA	pBBR-FlgL-gfp
FliE-F	AGGGAACAAAAGCTGGGTACCGATGAGCGACTCCGTTACCTCCATC	To generate
FliE-R	GAACAGTTCTTCACCTTTACTCATCAGCGGCATGTTCATGACGTCCTG	pBBR-FliE-gfp
MotA-F	AGGGAACAAAAGCTGGGTACCGGTGCTGGCGATCGTGGCGATCGTG	To generate
MotA-R	GAACAGTTCTTCACCTTTACTCATATGCAAGTAGCCTGCCAATTTCGA	pBBR-MotA-gfp
MotB-F	AGGGAACAAAAGCTGGGTACCGATGGCCCGTCGTCGCACACACCAC	To generate
MotB-R	GAACAGTTCTTCACCTTTACTCATCTGCGATGGCGGATCGGGTGCGAG	pBBR-MotB-gfp
FliG-F	AGGGAACAAAAGCTGGGTACCGGTGAAGCCTGAAACAGAACCGATG	To generate
FliG-R	GAACAGTTCTTCACCTTTACTCATTACCATCGCCTCCGCACCGGCGCC	pBBR-FliG-gfp
Le5109-F	CGACTTCAACACCAACGACA	To generate

Le5109-R	CGGCGGATCAGTTGTTTCTC	To test the transcription of Le5109
Le5111-F	GGTGTTGAACGACCTCAACC	To generate
Le5111-R	GTTCGCTCTCGATGAACTGC	To test the transcription of Le5111
Le5116-F	GACCACGCTGATGAAGTTCC	To generate
Le5116-R	GATGAAGTAGCCCGGGAAGA	To test the transcription of Le5116
Le5117-F	CGGTGTTCTCGATGCTCAAC	To generate
Le5117-R	CATTCCAGCGAGAAGATGCC	To test the transcription of Le5117
Le5118-F	GTTATTTCGCCCTGGTGTGG	To generate
Le5118-R	CGGCAGGTTGATCCAGAAGA	To test the transcription of Le5118
16s-F	ACGGTCGCAAGACTGAAACT	Quantitative Real-time PCR
16s-R	AAGGCACCAATCCATCTCTG	Quantitative Real-time PCR
lafB-F	CATCACATCATCTCCGATGC	Quantitative Real-time PCR
lafB-R	CAGTTCCACCTTCTCCTTGC	Quantitative Real-time PCR
lafB-F	CCGGAATTCAAAAGCTTGCGGAAAACCGCC	To generate
lafB-R	GCTCTAGAGCGTGACCGGGTCGGCGTTCG	pBX-*lafB*
FleQ-F	TAACTTTAAGAAGGAGATATACATATGAGTGAGTCCCGCATTCTGTTG	To generate
FleQ-R	GTGGTGCTCGAGTGCGGCCGCAAGCTTGTTGGCCAGCTCGGTCTGCTC	pET30A-FleQ
lafB-EMSA-F	ATTTTTTCCGGAGGGAAACG	To generate
lafB-EMSA-R	TTTCATTTTCTTTTTCCATT	*lafB* fragment
FleQ-pTRG-F	AAACCAGAGGCGGCCGGATCCATGAGTGAGTCCCGCATT	To generate

FleQ-pTRG-R	GCGCCAGCTCAGACTGAATTCTCAGTTGGCCAGCTCGGT	pTRG-FleQ

References

Fang X, Ahmad I, Blanka A, Schottkowski M, Cimdins A, Galperin MY, Romling U, Gomelsky M. 2014. GIL, a new c-di-GMP-binding protein domain involved in regulation of cellulose synthesis in enterobacteria. Mol Microbiol 93:439-452.

Harris LM, Merrick CJ. 2015. G-quadruplexes in pathogens: a common route to virulence control? PLoS Pathog 11:e1004562.

Qian G-l, Hu B-s, Jiang Y-h, Liu F-q. 2009. Identification and Characterization of Lysobacter enzymogenes as a Biological Control Agent Against Some Fungal Pathogens. Agricultural Sciences in China 8:68-75.
